# Metal Centers as Nucleophiles: Oxymoron of Halogen Bond‐Involving Crystal Engineering

**DOI:** 10.1002/chem.202103173

**Published:** 2021-10-29

**Authors:** Daniil M. Ivanov, Nadezhda A. Bokach, Vadim Yu. Kukushkin, Antonio Frontera

**Affiliations:** ^1^ Institute of Chemistry Saint Petersburg State University Universitetskaya Nab. 7/9 Saint Petersburg 199034 Russian Federation; ^2^ Institute of Chemistry and Pharmaceutical Technologies Altai State University Barnaul 656049 Russian Federation; ^3^ Department of Chemistry Universitat de les Illes Balears Palma de Mallorca Baleares 07122 Spain

**Keywords:** halogen bonding, noncovalent interactions, nucleophilic metal centers, σ-hole interactions, theoretical calculations

## Abstract

This review highlights recent studies discovering unconventional halogen bonding (HaB) that involves positively charged metal centers. These centers provide their filled *d*‐orbitals for HaB, and thus behave as nucleophilic components toward the noncovalent interaction. This role of some electron‐rich transition metal centers can be considered an oxymoron in the sense that the metal is, in most cases, formally cationic; consequently, its electron donor function is unexpected. The importance of Ha⋅⋅⋅*d*‐[M] (Ha=halogen; M is Group 9 (Rh, Ir), 10 (Ni, Pd, Pt), or 11 (Cu, Au)) interactions in crystal engineering is emphasized by showing remarkable examples (reported and uncovered by our processing of the Cambridge Structural Database), where this Ha⋅⋅⋅*d*‐[M] directional interaction guides the formation of solid supramolecular assemblies of different dimensionalities.

## Graphical abstract

**Metal centers** provide their filled *d*‐orbitals for halogen bonding, and behave as nucleophilic components toward the noncovalent interaction.

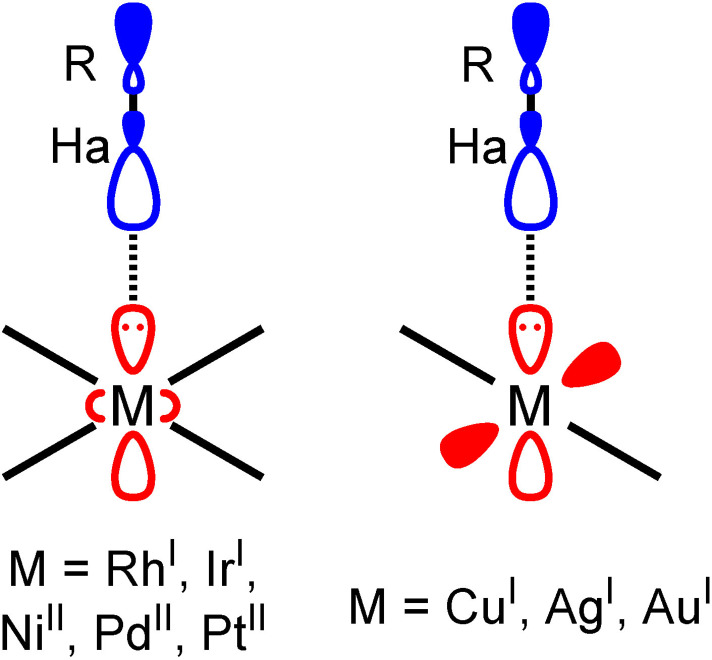

## 1 Introduction

Halogen bonding (HaB) is a representative (and likely the most actively studied) σ‐hole interaction.[^1^, ^2^, ^3^] The rapidly expanding body of literature on the subject, including reviews and book chapters, reflects a growing interest in HaB within the broad scientific community.[^4^, ^5^, ^6^, ^7^, ^8^] A number of theoretical investigations have been instrumental for understanding the halogen bond's directionality and strength.[^1^, ^2^, ^3^, ^4^, ^5^, ^6^, ^7^, ^8^, ^9^, ^10^, ^11^] This type of noncovalent interaction finds applications in supramolecular chemistry,[^4^, ^5^, ^6^, ^7^, ^8^, ^9^, ^10^, ^11^] catalytic transformations (including so‐called noncovalent catalysis),[^12^, ^13^, ^14^, ^15^, ^16^, ^17^, ^18^] synthetic coordination and organometallic chemistry,[^19^, ^20^, ^21^, ^22^, ^23^, ^24^] polymer chemistry,^[25]^ and drug discovery.[^26^, ^27^, ^28^, ^29^] Importantly, HaB functions in human physiology, and its role has been uncovered in reviews on this subject.[^30^, ^31^]

HaB is probably best known for its extensive use in crystal engineering[^32^, ^33^] because HaB is more directional than the more common hydrogen bonding, allowing for designs of solid‐state architectures with different dimensionality. Most studies of HaB use heavier halogens Br and I as σ‐hole donors, exhibiting substantial polarizabilities[^34^, ^35^] bonded to electron withdrawing groups (EWGs). Commonly used HaB acceptors (electron donors) are lone pair (LP)‐bearing atoms, such as halogens and lighter chalcogens and pnictogens. Furthermore, electron‐rich C atoms (for example, in carbenes R_2_C:,^[36]^ isocyanides RNC:,^[37]^ carbon monoxide OC:,^[38]^ and anionic species such as alkyl carbanions C(sp^3^)^−[39]^) and π‐systems (for example, −C=C−, −C≡C−, arene)[^40^, ^41^, ^42^] have also been recognized as good HaB acceptors.

**Scheme 1:**
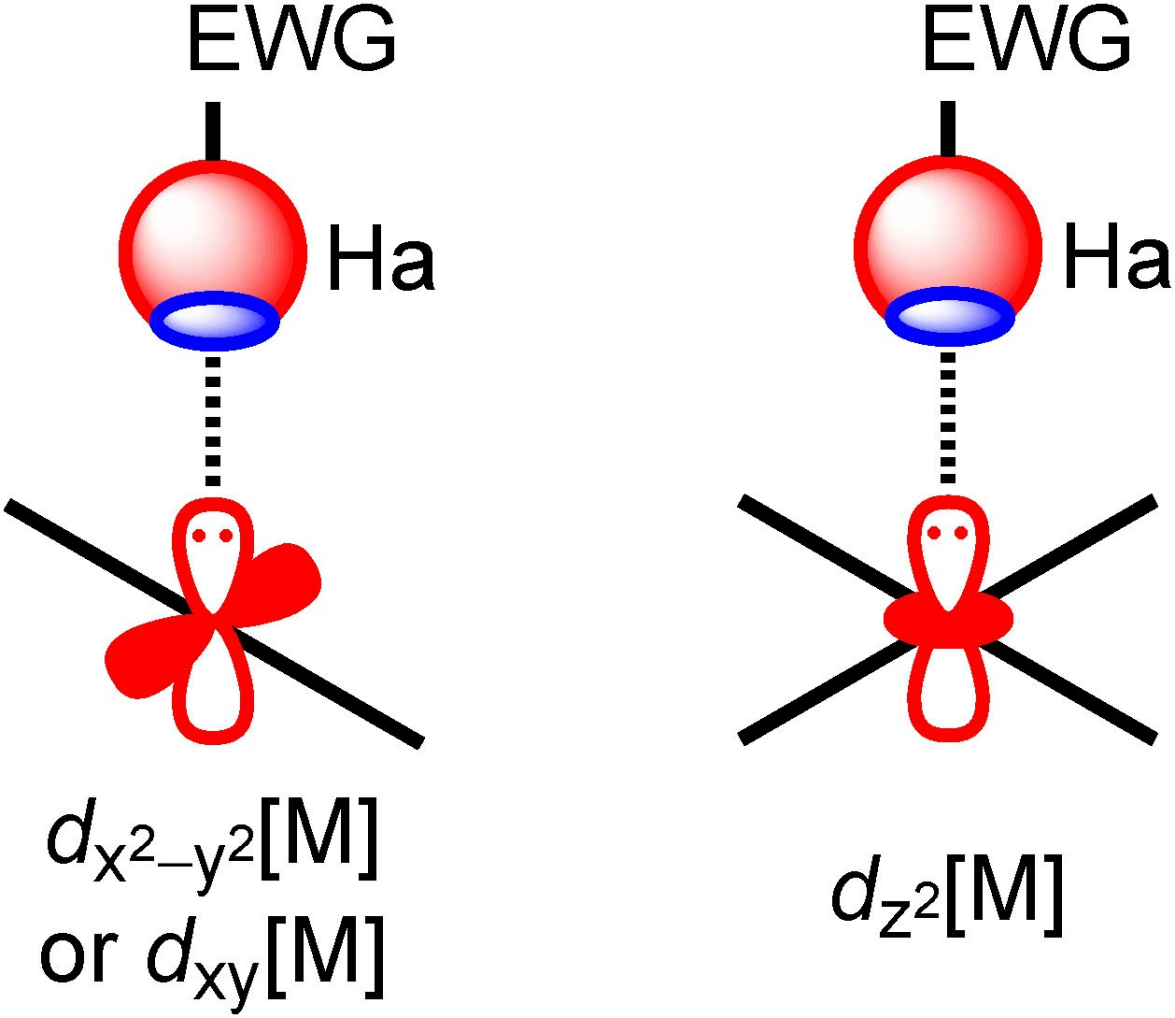
An unconventional metal‐involving σ‐(Ha)‐hole⋅⋅⋅*d*[M] halogen bonding: Groups 9–10 (left panel) and Group 11 (right panel) metal centers.

In contrast, HaB‐based crystal engineering involving the utilization of a filled *d*‐orbital in a positively charged metal center functioning as a HaB acceptor remains uncommon; the nucleophilicity of positively charged metal centers is likely to be perceived as an oxymoron by most chemists. However, it has been proven that a metal can act as an electron donor if it contains at least one sterically available lone pair that can interact with the empty σ*‐orbital (R−Ha) of the halogen (see Scheme 1). In recent years, only a small number of studies have utilized such unconventional HaBs in crystal engineering and supramolecular chemistry.[^43^, ^44^]

This highlight primarily focuses on this metal‐involving type of HaB, emphasizing its relevance in solid‐state, Group 9 (Rh, Ir), 10 (Ni, Pd, Pt), or 11 (Cu, Au) metal‐based cocrystals with typical HaB donors (see Scheme 1). In addition to the description and discussion of the geometric features of the Ha⋅⋅⋅*d*‐[M] HaBs in the solid‐state, further rationalization of the interactions using molecular electrostatic potential surfaces and a QTAIM/NCIplot combined analysis is also provided. These computational tools provide a convenient confirmation of the HaB nature of the interaction and the nucleophilicity of the metal centers.

## 2 σ‐Hole⋅⋅⋅M Interactions

First, we performed a Cambridge Structural Database (CSD) search for structures featuring short X⋅⋅⋅M (X=Cl, Br, I; M=any metal) contacts whose parameters do not contradict the IUPAC criteria of HaB;^[45]^ the obtained results for >100 structures are gathered in Table S1 (see Supporting Information). For a significant number of structures, these contacts were not reported in corresponding publications and, for another large, massive structure, these contacts were only mentioned; their nature and characteristics were not analyzed. Only in some rare instances, predominantly in the recent works by our group, were the X⋅⋅⋅M contacts verified by experimental physicochemical methods and additionally supported by theoretical calculations.

As follows from the inspection of the data of Table S1, metal centers are mainly presented by late transition metals with *d* ^8^ (Rh^I^, Ni^II^, Pd^II^, and Pt^II^) and *d* ^10^ (Ag^I^, Au^I^) electron configurations. Within the same group of elements, as a common trend, heavier transition metals demonstrate a greater tendency to form X⋅⋅⋅M contacts than lighter metals, for example, Au^I^ versus Cu^I^ and Pt^II^ versus Ni^II^. In some instances, for cocrystals including I_2_ as a HaB donor, halogen⋅⋅⋅halogen interactions that are intermediate between noncovalent and covalent were observed (Entries 1 and 54–56).

Apart from single‐crystal X‐ray diffractometry (XRD), some other methods – namely, NMR, UV‐vis, photoluminescence spectroscopies, cyclic voltammetry and conductance measurements – have been used for the indirect identification of X⋅⋅⋅M HaBs (Table S2).

Along with experimental studies, a significant number of theoretical studies have focused on metal‐involving HaB (Table S3). Most of these reports used single‐point “quasi‐solid‐state” DFT calculations,[^46^, ^47^] while examples of Kohn‐Sham calculations with periodic boundary conditions are still quite rare.^[47]^ Many studies exclusively use the QTAIM method for revealing HaB,[^44^, ^48^] as recommended in the IUPAC definition of HaB. However, recently the QTAIM approach has been combined with other approaches, such as the NCIPlot analysis.[^46^, ^49^] Predominantly, XRD structure coordinates were employed for subsequent theoretical studies,[^46^, ^48^, ^50^] while in some instances the theoretical approaches used only model complexes, for example, I⋅⋅⋅Co(I) and I⋅⋅⋅Au(−1).[^51^, ^52^, ^53^]

In the following subsections, we inspected the appropriate geometrical parameters and computationally analyzed the XRD structures involving structure‐directing σ‐hole⋅⋅⋅*d*[M] interactions for the occurrence of HaBs and highlighted applications of these interactions for HaB‐involving crystal engineering.

### 2.1 σ‐Hole⋅⋅⋅d^10^[M]

Sterically accessible potentially nucleophilic metal centers can have a *d* ^10^‐subshell in linear complexes and a *d* ^8^‐subshell in square‐planar complexes. In the case of *d* ^10^‐gold(I) and *d* ^10^‐copper(I) in linear complexes, *d_xy_*‐ and *d_x_* ^2^_‐*y*_^2^‐orbitals are sterically unhindered and available to function as electron donors/HaB acceptors.

#### 2.1.1 Gold

Comprehensive theoretical studies have demonstrated that gold in the +1 oxidation state (for example, formally positively charged) can still function as a Lewis base and, particularly, as an excellent HaB acceptor.[^48^, ^53^, ^54^] Depending on the nature of the ligands, the strength of HaB, involving I_2_ as a σ‐hole donor, ranges from −46.3 kcal/mol to −5.9 kcal/mol for the anionic (*n*=−1) or cationic (*n*=+1) [(Y−Au‐Y)⋅⋅⋅I_2_]^*n*^ adduct.

Although predominantly unnoticed by the original authors, we found examples of solid‐state structures that demonstrate I⋅⋅⋅Au^[55]^ and Cl⋅⋅⋅Au^[54]^ contacts.^[56]^ These gold‐involving interactions with halogens were not attributed to HaBs, most likely because the Au center is formally positively charged; hence, it was considered a Lewis acid. Expectedly, no appropriate theoretical calculations were performed. In this review, we provide our computational data confirming the availability of X⋅⋅⋅Au interactions and uncovering their nature.

**Figure 1:**
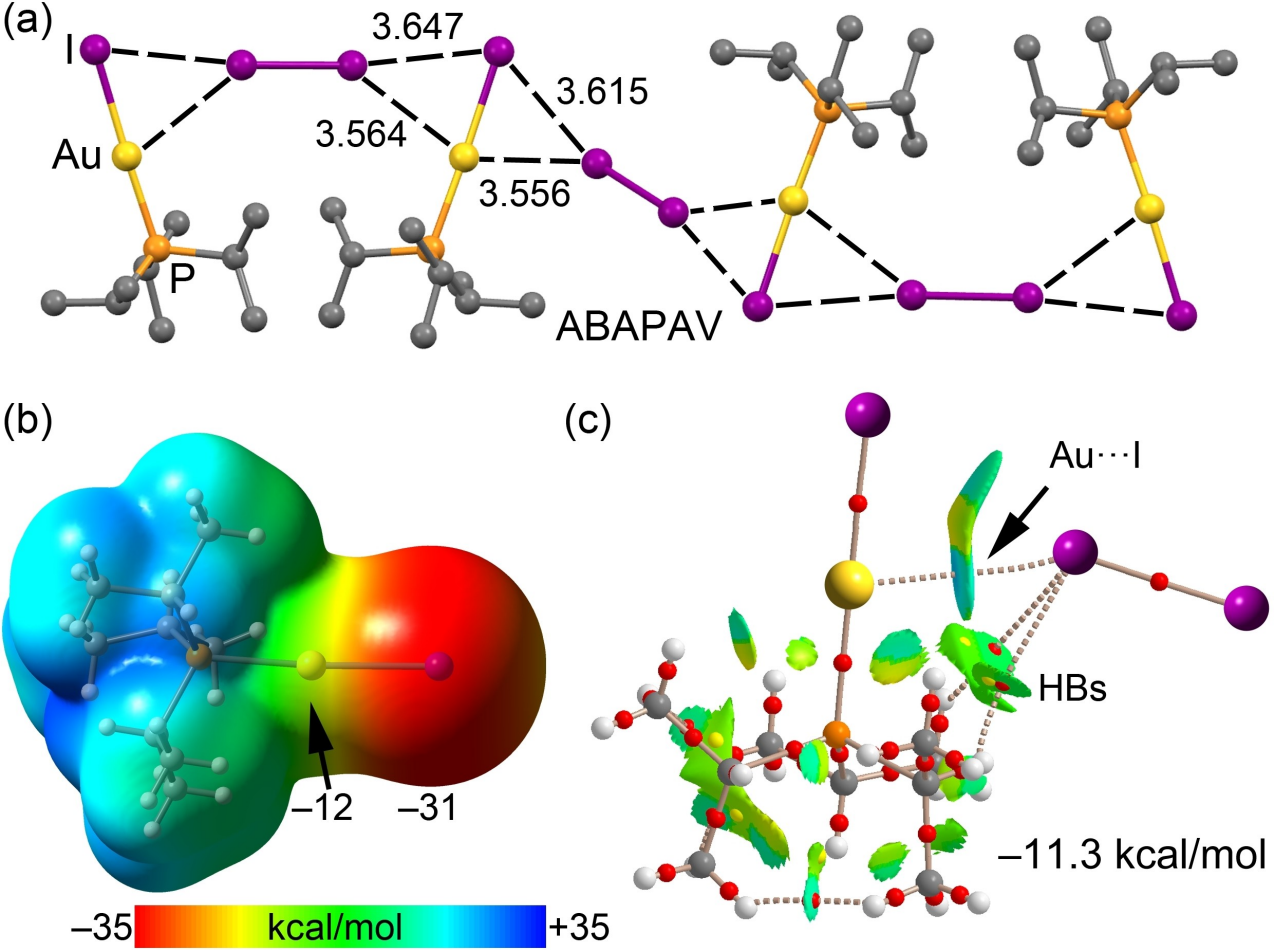
(a) Partial view of the XRD structure CSD refcode ABAPAV^14^ showing the 1D assembly governed by bifurcated I⋅⋅⋅I,*d_z_* ^2^[Au] interactions; (b) MEP surface of [AuI(PPr^*i*^)_3_] using the 0.001 a.u. The MEP values at selected points of the surface are indicated; (c) combined QTAIM distribution of CPs (bond CPs in red and ring CPs in yellow) and bond paths with the NCIplot surfaces (isosurface=0.5 a.u., gradient cutoff=0.4 a.u., color scale −0.03 a.u.≤sign(*λ*_2_)*ρ*≤0.03 a.u.). Hereinafter distances are given in Å and the interaction energies computed using the PBE0‐D3/def2‐TZVP level of theory.

In Figure 1(a), we show the XRD structure of a selected example, where the [AuI(PPr^*i*^)_3_] entities are interconnected by I_2,_ forming 1D assemblies. The I atom of I_2_ points to approximately the middle of the Au−I bonds, thus establishing bifurcated I⋅⋅⋅I,*d*_z_^2^[Au] HaB interactions. Interestingly, the I⋅⋅⋅Au distance is shorter than the I⋅⋅⋅I distance. An analysis of the MEP surface plot (Figure 1b) confirms that the Au center is nucleophilic rather than electrophilic (−12 kcal/mol) and reveals that the MEP minimum is located at the I atom (−31 kcal/mol). The QTAIM analysis of a dimer extracted from the XRD structure, the dimer with the shortest I⋅⋅⋅Au distance, revealed the I⋅⋅⋅Au contact. The latter is characterized by a bond critical point (CP) and a bond path connecting I to the Au atom. Curiously, there is no bond CP interconnecting both I atoms. The NCIplot index analysis evidences the attractive nature of the interaction (blue isosurface) and demonstrates the existence of an I⋅⋅⋅I contact characterized by a green isosurface located between both atoms. The comparison of the NCIPlot colors at the I⋅⋅⋅Au and I⋅⋅⋅I contacts shows that Au⋅⋅⋅I is stronger. The location of the interacting I atom of I_2_ is also affected by the presence of two ancillary H‐bonds involving the H atoms of the Pr^*i*^ groups (Figure 1c). Therefore, the H‐bonds and the I⋅⋅⋅Au HaB compete with the more electrostatically‐favored I⋅⋅⋅I interaction. The interaction energy of the dimer is moderately strong (−11.3 kcal/mol) in line with the NCI plot analysis and the cooperative participation of several interactions.

**Figure 2:**
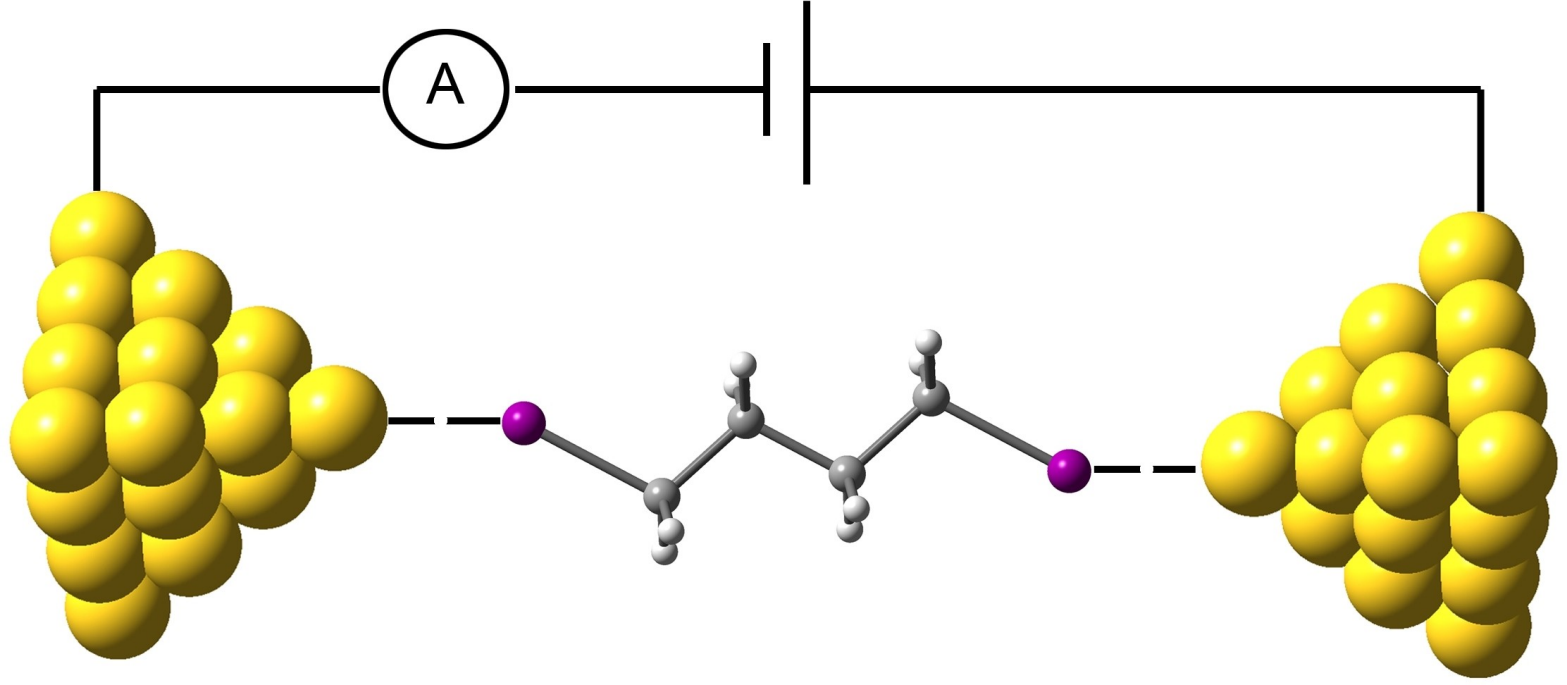
Schematic diagram of a scanning tunneling microscopy break junction (STM‐BJ) and the I⋅⋅⋅Au interaction with butane‐1,4‐diiode. Adapted from Ref. [60,61].

Further *indirect* evidence favoring I⋅⋅⋅Au HaBs has been reported in the literature: (i) iodoperfluorobenzenes and gold nanoparticles form HaB in water solutions[^57^, ^58^, ^59^] and (ii) iodine‐terminated alkanes with Au tips form I⋅⋅⋅Au, as verified by scanning tunneling microscopy break junction (STM‐BJ) and single molecular conductance measurements (Figure 2).[^60^, ^61^]

In the end of this section, we would like to emphasize that I*⋅⋅⋅*Ag,[^62^, ^63^] Br*⋅⋅⋅*Ag,^[64]^ and Cl*⋅⋅⋅*Ag^[65]^ short contacts can also be regarded as *d* ^10^‐metal‐involving HaB on consideration of their almost linear R−X*⋅⋅⋅*Ag angles (for details see Table S1). However, appropriate theoretical calculations are required to support this hypothesis.

### 2.2 σ‐Hole⋅⋅⋅d^8^[M]

#### 2.2.1 Nickel(II)

**Figure 3:**
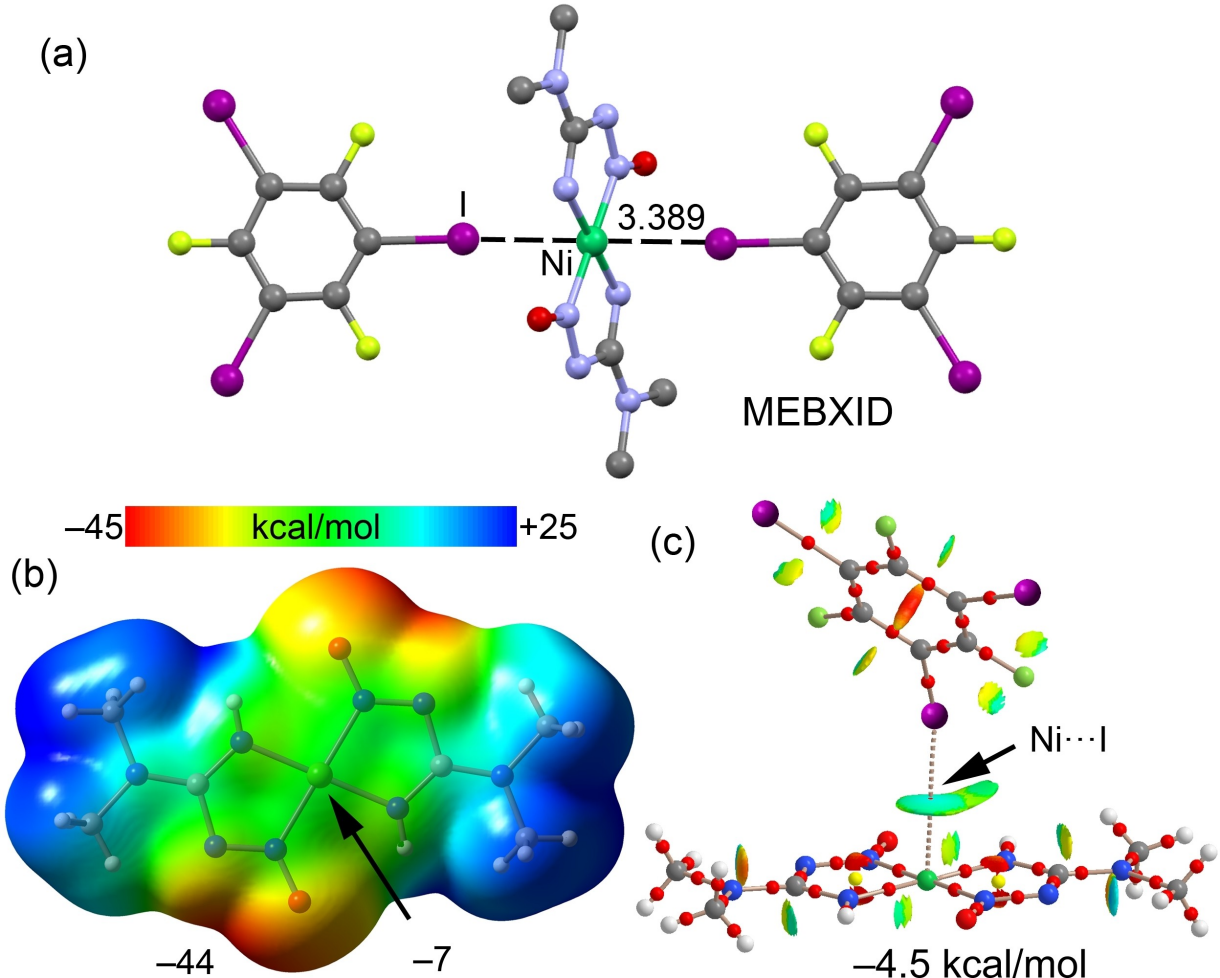
(a) Partial view of the XRD structure of CSD refcode MEBXID^[44]^ showing the supramolecular assembly governed by I⋅⋅⋅*d*_z_^2^[Ni^II^] interactions; (b) MEP surface of (nitrosoguanidinate)_2_Ni^II^ complex using the 0.001 a.u. The MEP values at selected points of the surface are indicated; (c) combined QTAIM distribution of CPs (bond CPs in red and ring CPs in yellow) and bond paths with the NCIplot surfaces (isosurface = 0.5 a.u., gradient cutoff = 0.4 a.u., color scale −0.03 a.u. ≤ sign(*λ*_2_)*ρ* ≤ 0.03 a.u.). The dimerization energy is indicated.

The ability of nickel(II) square‐planar complexes to interact with iodine derivatives has been studied in terms of electrophilic‐nucleophilic dualism.^[44]^ Actually, three types of I⋅⋅⋅Ni contacts have been verified upon the examination of XRD structures in the CSD, namely, (i) Ni⋅⋅⋅I semicoordination of the electrophilic nickel(II) center with an electron belt of I, (ii) metal‐involving HaB between iodine and the nucleophilic nickel(II)‐*d*_z_^2^ center,[^44^, ^50^] and (iii) the boundary case, where it is difficult to differentiate between the semicoordination bond and HaB due to the directionality of the C−I⋅⋅⋅Ni interaction. One example of a metal‐involving HaB is further described herein. Figure 3(a) shows the XRD structure of a (nitrosoguanidinate)Ni^II^ complex that establishes symmetrically related I⋅⋅⋅Ni interactions with co‐crystallized 1,3,5‐triiodotrifluorobenzene (FIB). The distance is significantly shorter than the sum of the Bondi van der Waals radii (*Σ*_vdW_; Ni+I=3.61 Å). The ∠C−I⋅⋅⋅Ni angle is far from linear (142.5°) and far from the ideal orientation expected for a semicoordination bond (∼90°). The MEP surface plot (Figure 3b) shows that the MEP is negative over the central Ni atom and that the minimum is found in the molecular plane between the N and O atoms of nitrosoguanidinate. The existence and attractive nature of the I⋅⋅⋅Ni contact is corroborated by the QTAIM/NCIplot analysis shown in Figure 3(c), which reveals a bond CP interconnecting the I and Ni atoms and a bluish isosurface between them. Moreover, the dimerization energy is −4.5 kcal/mol that is in the range of recent reports on σ‐hole⋅⋅⋅*d*_z_^2^[M] HaBs.^[46]^ The value of MEP at Ni indicates that this interaction has a predominant HaB nature. This was further supported by a recent study,^[67]^ which has evidenced that the σ‐hole at the I‐atom in FIB embraces a large region and can be involved in HaBs with C−I⋅⋅⋅:A angles up to 105°.

**Figure 4:**
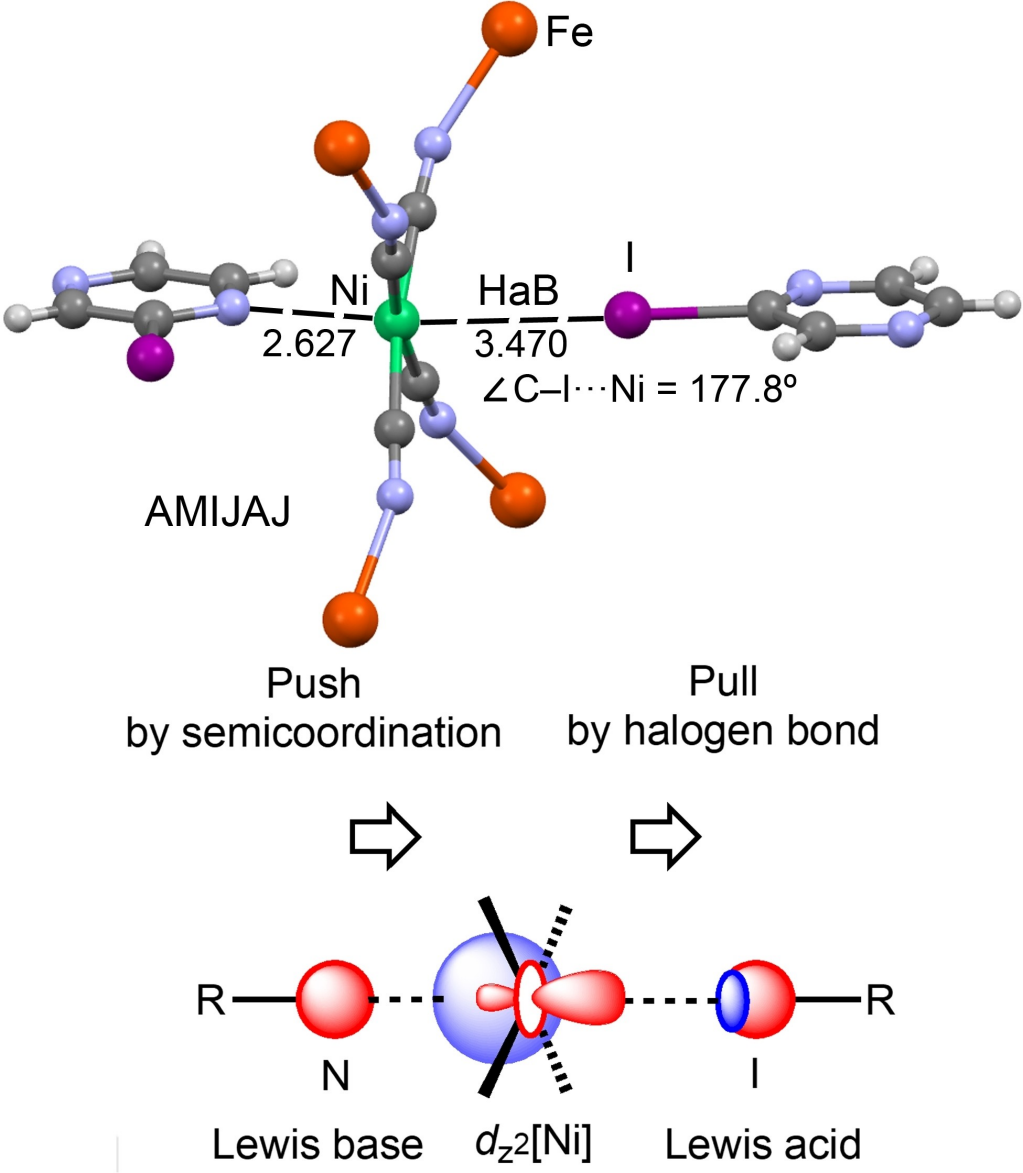
Partial view of the XRD structure of CSD refcode AMIJAJ^[66]^ (top panel) and explanatory scheme illustrating the push‐pull effect (bottom panel).

Figure 4 shows an additional example of a directional HaB interaction involving the Ni^II^ atom as an electron donor/HaB acceptor.^[66]^ In the XRD structure of Fe(2‐iodopyrazine)(H_2_O)Ni(CN)_4_, the I⋅⋅⋅Ni distance is quite short (3.470 Å; *Σ*_vdW_ is 3.61 Å), and the ∠(C−I⋅⋅⋅Ni) angle (177.8°) is very close to linearity, thus evidencing that the σ‐hole is pointing to the *d_z_* ^2^[Ni^II^] orbital.

A push‐pull effect was proposed in AMIJAJ, where the *N* atom of pyrazine donates electron charges to nickel(II), polarizing the *d*_z_^2^ orbital toward the I atom and providing a synergetic pull effect on this orbital (Figure 4). This effect is synergetic for both metal‐involving HaB and the semicoordination in AMIJIR (structurally similar Pd complex) and AMIJEN (structurally similar Pt complex).

Two other structures, namely, AMIJIR with Pd^II^ and AMIJEN with Pt^II^,^[66]^ are isostructural to the nickel(II)‐based structure AMIJAJ depicted in Figure 4, and the former two structures display highly directional HaB interactions with the positively charged Pt or Pd centers, acting as *d_z_* ^2^ electron donors. The I⋅⋅⋅M distances are comparable (Pd: 3.498, Pt: 3.467 Å) to the I⋅⋅⋅Ni separation, and the ∠(C−I⋅⋅⋅Pd) angles (177.6 and 177.1°, respectively) provide evidence that the σ‐hole is directed toward the *d_z_* ^2^[M^II^] orbital.

#### 2.2.2 Rhodium(I)

**Figure 5:**
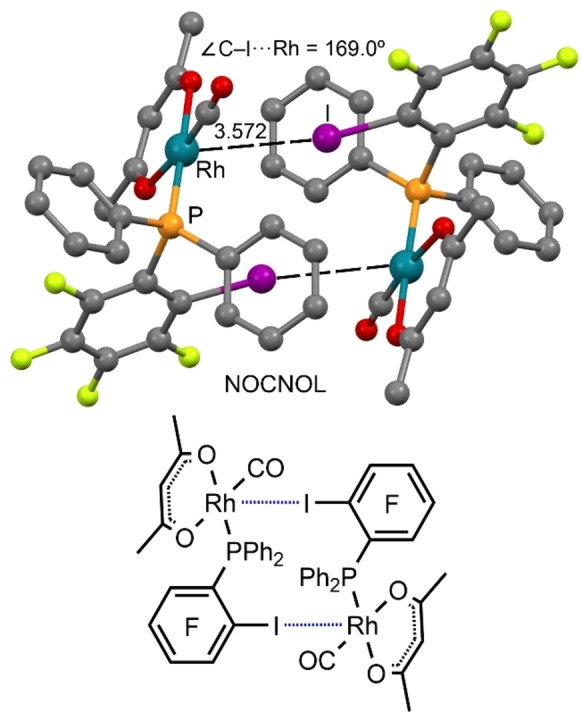
Partial view of CSD structure NOCNOL.^[69]^ HaBs are given by dashed lines, while H atoms were omitted for clarity.

Until very recently, short contacts between an Rh^I^ center and an iodine atom were identified and attributed to the category of coordinative bonding.[^52^, ^68^] A CSD inspection reveals a unique structure (CSD refcode: NOCNOL)^[69]^ showing directional C−I⋅⋅⋅*d*_z_^2^[Rh^I^] contacts that were out of scope of the corresponding papers. This compound (Figure 5) forms self‐assembled dimers in the solid‐state featuring two symmetrically equivalent C−I⋅⋅⋅*d*_z_^2^[Rh] interactions. The I⋅⋅⋅Rh distance is significantly shorter than the sum of vdW radii (4.00 Å), considering the crystallographic Batsanov radius (2.00 Å) for Rh.^[70]^ The directionality of the interaction (∠C−I⋅⋅⋅Rh=169.0°) along with the electron withdrawing nature of the arene indicate that this interaction can be defined as a HaB.

We recently reported that the two rhodium(I) complexes [RhX(COD)]_2_ (X=Cl, Br; COD=1,5‐cyclooctadiene) form co‐crystals with several σ‐hole iodine(I)‐based donors.^[46]^ XRD studies, in combination with extensive theoretical considerations, revealed that the *d_z_* ^2^ orbitals of two positively charged rhodium(I) centers provide sufficient nucleophilicity to form three‐center HaB with σ‐(X)‐hole donors.

**Figure 6:**
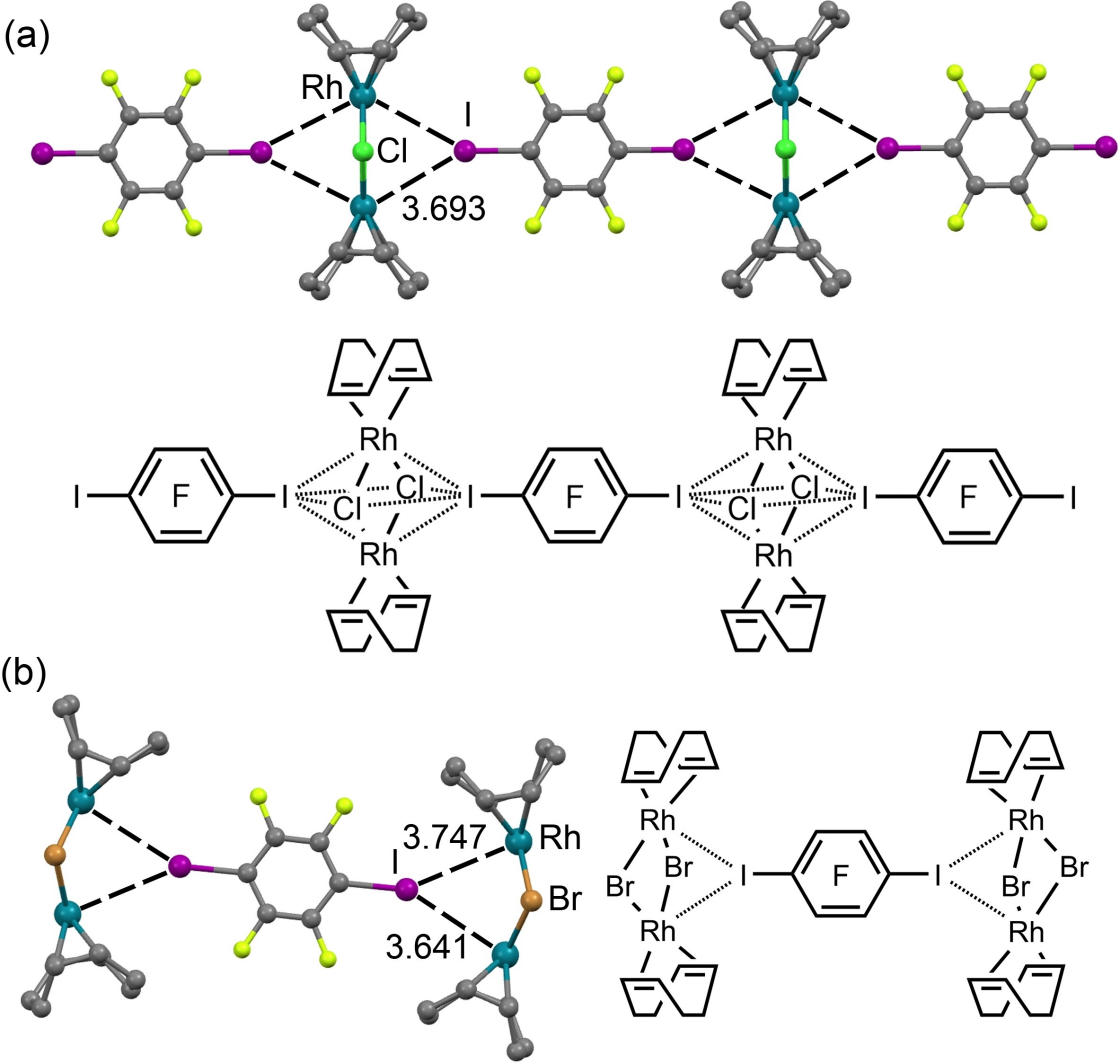
Partial view of the XRD structures of [RhCl(COD)]_2_ ⋅ (C_6_F_4_I_2_) (a) and [RhBr(COD)]_2_ ⋅ (C_6_F_4_I_2_) (b).

The two metal centers function as an integrated HaB acceptor, providing assembly via Rh^I^‐involving HaB to give, in particular, 1D supramolecular arrays. In the [RhCl(COD)]_2_ ⋅ (C_6_F_4_I_2_) structure (Figure 6a), two iodine σ‐hole donors interact with the Rh_2_Cl_2_ core of the complex above and below the Rh_2_Cl_2_ plane (the core is perfectly planar). In contrast, the crystal structure of the cocrystal [RhBr(COD)]_2_ ⋅ (C_6_F_4_I_2_) (Figure 6b) shows a distortion of the Rh_2_Br_2_ entity, where the Rh centers are tilted toward the σ‐(I)‐hole, facilitating the formation of two C−I⋅⋅⋅*d*_z_^2^[Rh^I^] interactions.

**Figure 7:**
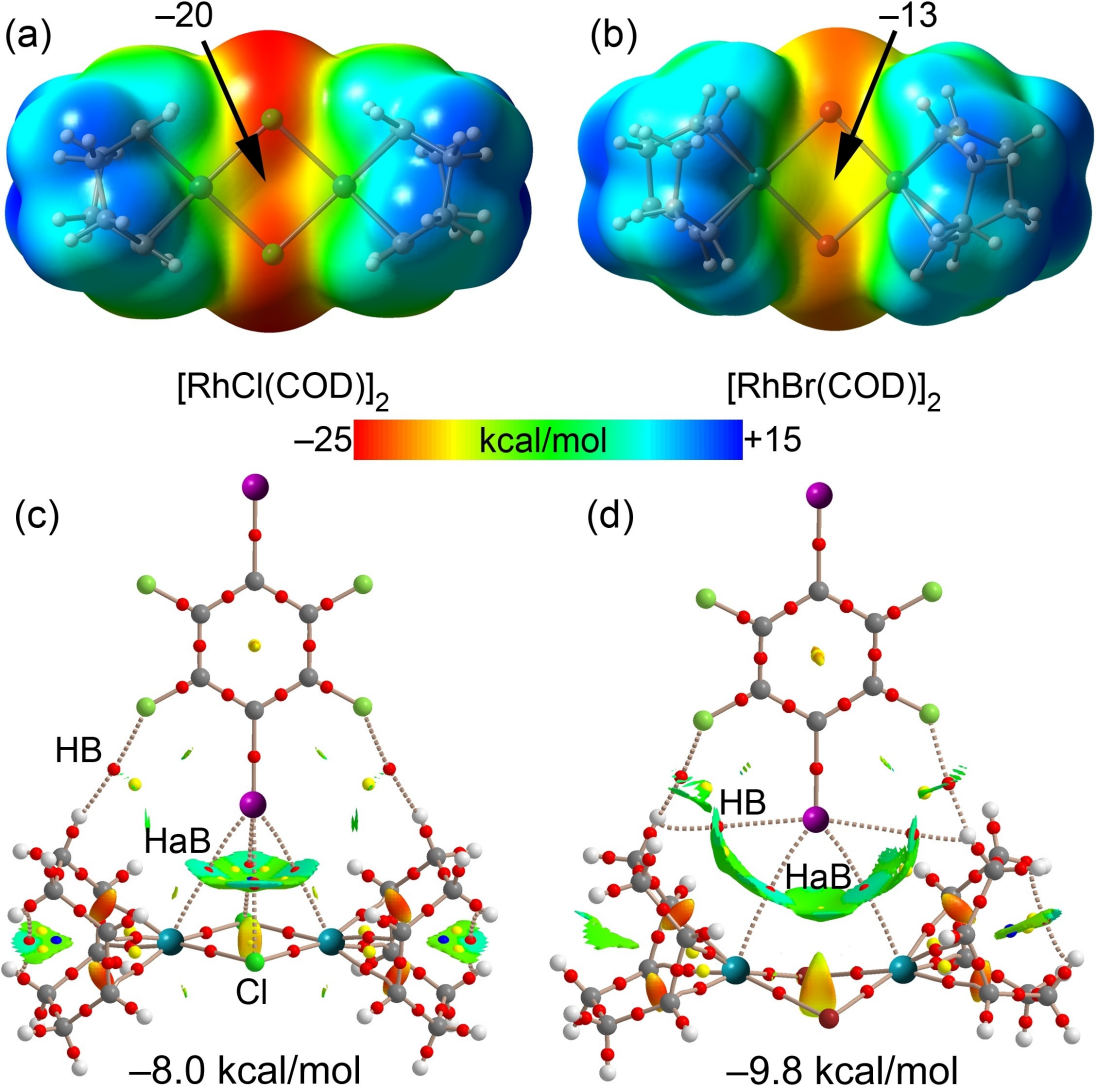
MEP surfaces of [RhCl(COD)]_2_ (a) and [RhBr(COD)]_2_ (b) using the 0.001 a.u. The MEP values at selected points of the surfaces are indicated. Combined QTAIM distribution of CPs (bond CPs in red and ring CPs in yellow) and bond paths with the NCIplot surfaces (isosurface = 0.5 a.u., gradient cutoff = 0.4 a.u., color scale −0.03 a.u. ≤ sign(*λ*_2_)*ρ* ≤ 0.03 a.u.) for [RhCl(COD)]_2_⋅(C_6_F_4_I_2_) (c) and [RhBr(COD)]_2_⋅(C_6_F_4_I_2_) (d). The dimerization energies are indicated.

The MEP surfaces represented in Figure 7 (top panel) confirm the nucleophilicity of the Rh_2_Cl_2_ and Rh_2_Br_2_ cores (−20 and −13 kcal/mol). The combined QTAIM/NCIPlot analysis of [RhCl(COD)]_2_ ⋅ (C_6_F_4_I_2_) (Figure 7c) reveals an intriguing feature that is a unique *tetrafurcated* Ar^F^(*μ*_4_‐I)⋅⋅⋅[Rh_2_Cl_2_] short interaction, where the iodine σ‐hole interacts with all four atoms of the Rh_2_Cl_2_ moiety (four bond CPs connect the I‐atom to the core). This unprecedented interaction, also confirmed by the NCIplot isosurface, is responsible for the propagation of the 1D‐chain ⋅⋅⋅[Rh_2_Cl_2_]⋅⋅⋅I(C_6_F_4_)I⋅⋅⋅[Rh_2_Cl_2_]⋅⋅⋅ shown in Figure 6(a).

The QTAIM analysis of the cocrystal [RhBr(COD)]_2_ ⋅ (C_6_F_4_I_2_) (Figure 7d) revealed that the I atom is connected to both Rh atoms, thus establishing bifurcated, instead of *tetrafurcated*, Ar^F^(μ_2_‐I)⋅⋅⋅[Rh,Rh] short contacts, thus resulting in discrete 2 : 1 supramolecular clusters (Figure 6b).

The interaction energies are moderately strong, ranging from −8.0 kcal/mol in X = Cl to −9.8 kcal/mol in X=Br, disclosing that the bifurcated binding mode is more favorable than the *tetrafurcated* one.

#### 2.2.3 Palladium(II)

**Figure 8:**
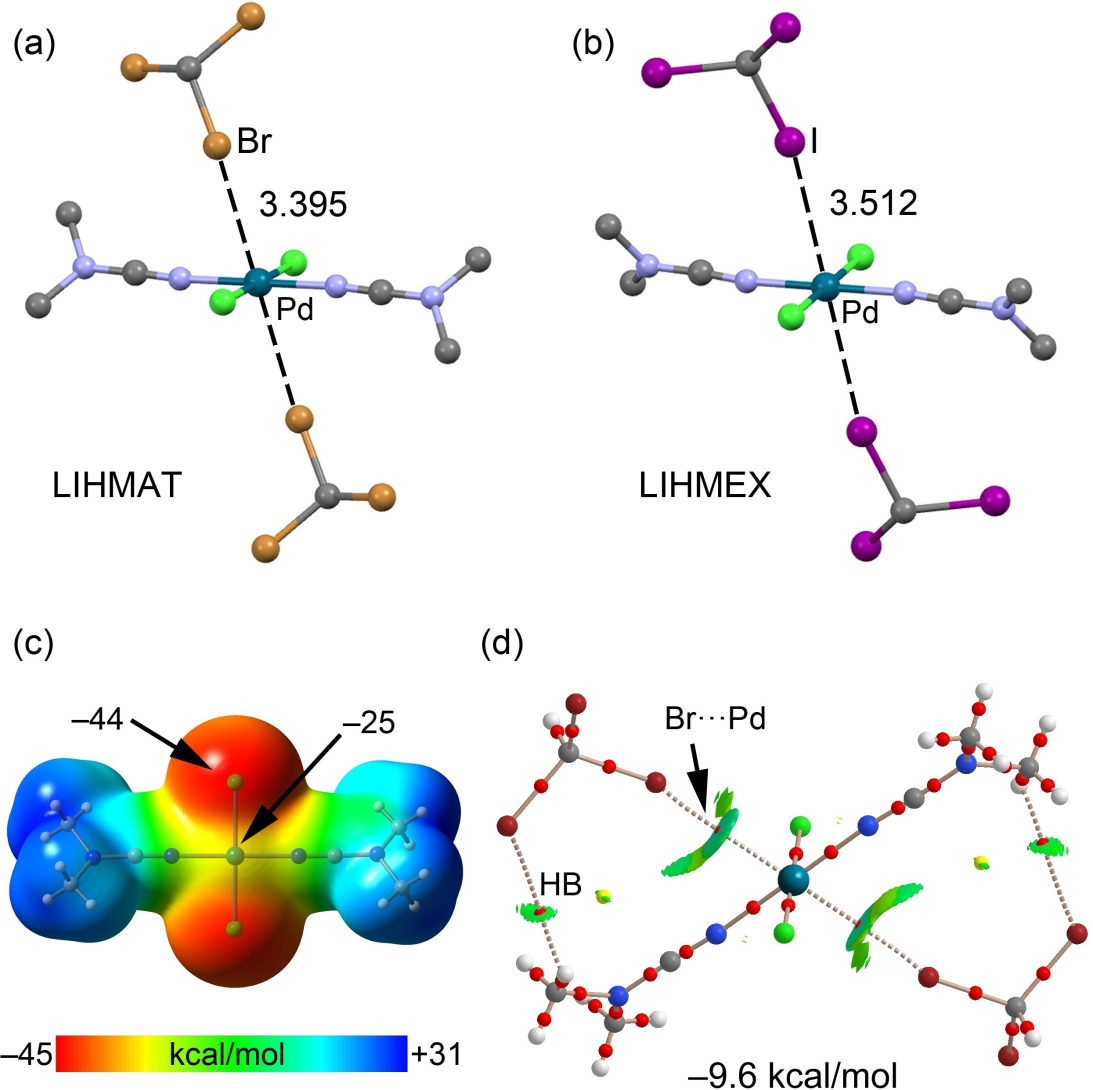
Top: Partial view of the XRD structures of CSD refcode LIHMAT^[71]^ (a) and LIHMEX^[71]^ (b) showing the supramolecular trimers governed by Br,I⋅⋅⋅*d*_z_^2^[Pd^II^] interactions. (c) MEP surface of the *trans*‐[PdCl_2_(NCNMe_2_)_2_] complex using 0.001 a.u. The MEP values at selected points of the surface are indicated. (d) Combined QTAIM distribution of CPs (bond CPs in red and ring CPs in yellow) and bond paths with the NCIplot surfaces (isosurface = 0.5 a.u., gradient cutoff = 0.4 a.u., color scale −0.03 a.u. ≤ sign(*λ*_2_)*ρ* ≤ 0.03 a.u.) for trimer assembly *trans*‐[PdCl_2_(NCNMe_2_)_2_] ⋅ 2CHBr_3_. The formation energy of the LIHMAT trimer is also indicated.

It has been reported that square‐planar palladium(II) complexes with chloride as ligands have an interesting ability to form bifurcated HaBs Br/I⋅⋅⋅*d_z_* ^2^[Pd^II^] that simultaneously involve the halide and a Pd^II^ center functioning as an integrated HaB acceptor.[^50^, ^71^, ^72^, ^73^, ^74^, ^75^, ^76^, ^77^, ^78^] In particular, the crystal structures of *trans*‐[MCl_2_(NCNMe_2_)_2_] ⋅ 2CHX_3_ (M=Pd, Pt; X=Br, I) were analyzed; two of which are shown in Figure 8(a, b). It is clear that the C−Br (LIHMAT) and C−I (LIHMEX) bonds point to the positively charged palladium(II) center. These contacts were attributed to HaBs by inspecting both the experimental data and the theoretical calculation data. In fact, the MEP surface shown in Figure 8(c) shows that the MEP value above and below the Pd^II^ center is negative (−25 kcal/mol) and thus could function as a σ‐hole acceptor.

Moreover, the QTAIM analysis of *trans*‐[PdCl_2_(NCNMe_2_)_2_] ⋅ 2CHBr_3_ (Figure 8d) shows a bond CP and a bond path connecting the Br atoms to the Pd center, thus confirming the existence of this contact. The attractive nature of the interaction is evidenced by the NCIplot isosurface, which shows that the isosurface extends toward the ligands, suggesting that the Pd‐bonded atoms from the ligands also participate in the binding mechanism. The interaction energy of the trimer is −9.6 kcal/mol, suggesting that each Br⋅⋅⋅*d_z_* ^2^[Pd^II^] is −4.8 kcal/mol, very similar to the I⋅⋅⋅*d_z_* ^2^[NI^II^] halogen bond commented above (Figure 3).

**Figure 9:**
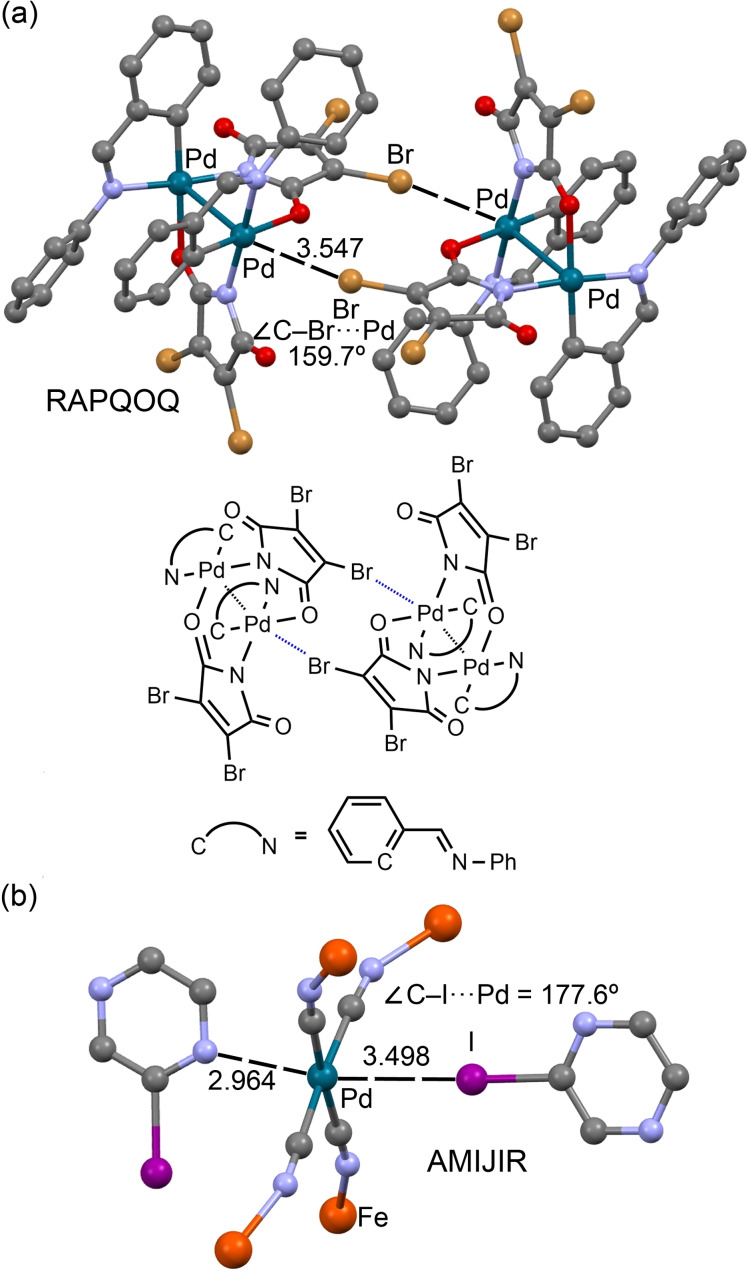
Partial view of the XRD structures of CSD refcodes RAPQOQ^[79]^ (a) and AMIJIR^[66]^ (b) showing the Br,I⋅⋅⋅*d_z_* ^2^[Pd] interactions. H‐atoms were omitted for clarity.

To further illustrate the importance of HaB involving *d*_z_^2^[Pd] acting as an electron donor, two additional XRD structures are shown in Figure 9. The dinuclear palladacyclic compound RAPQOQ^[79]^ forms self‐assembled dimers in the solid‐state, where two symmetrically equivalent C−Br⋅⋅⋅*d*_z_^2^[Pd] HaB interactions were identified. The short Pd⋅⋅⋅Pd distance (3.012 Å) likely increases the nucleophilicity of the outer orbitals, thus facilitating the formation of HaBs with σ‐hole Br donors (Figure 9a).

The second structure (AMIJIR)^[66]^ is isostructural to that commented above for nickel(II)‐based structure CSD refcode AMIJAJ (Figure 4) and shows a highly directional HaB interaction involving the Pd atom functioning as an HaB acceptor. The I⋅⋅⋅Pd distance is slightly longer (3.498 Å) than the I⋅⋅⋅Ni separation, and the ∠(C−I⋅⋅⋅Pd) angle (177.6°) shows that the σ‐hole points to the *d_z_* ^2^[Pd^II^] orbital. The push‐pull mechanism in AMIJAJ and AMIJIR structures (Section 2.2.1.), where the N atom of pyrazine donates an electron charge to nickel(II), polarizing the *d_z_* ^2^ orbital toward the I atom, is conceptually equivalent to the effect of the *d_z_* ^2^[Pd]⋅⋅⋅*d_z_* ^2^[Pd] interaction in RAPQOQ polarizing the outer part of the *d_z_* ^2^ orbital.

#### 2.2.4 Platinum(II)

**Figure 10:**
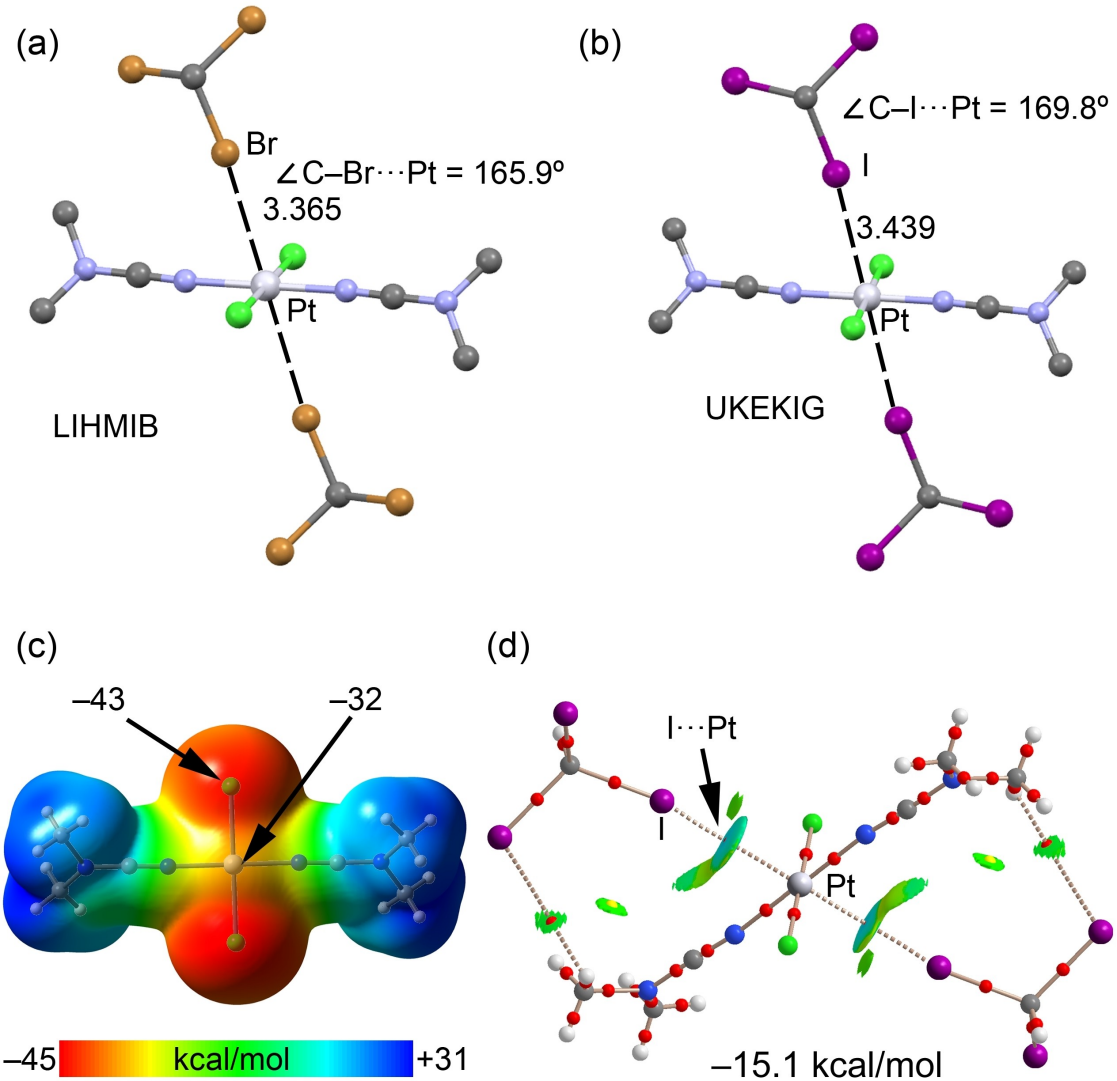
Partial view of the XRD structures of CSD refcode LIHMMIB^[71]^ (a) and UKEKIG^[81]^ (b) showing the supramolecular trimers governed by Br,I⋅⋅⋅*d_z_* ^2^[Pt] interactions. (c) MEP surface of the *trans*‐[PtCl_2_(NCNMe_2_)_2_] complex using 0.001 a.u. The MEP values at selected points of the surface are indicated. (d) Combined QTAIM distribution of CPs (bond CPs in red and ring CPs in yellow) and bond paths with the NCIplot surfaces (isosurface = 0.5 a.u., gradient cutoff = 0.4 a.u., color scale −0.03 a.u. ≤ sign(*λ*_2_)*ρ* ≤ 0.03 a.u.) for trimer assembly *trans*‐[PtCl_2_(NCNMe_2_)_2_] ⋅ 2CHI_3_. The formation energy of the UKEKIG trimer is also indicated.

Several examples of Pt^II^‐involved HaBs have been found and discussed in the literature, thus demonstrating the ability of platinum(II) square‐planar complexes to participate in this type of metal‐involving unconventional HaB.[^43^, ^47^, ^49^, ^50^, ^71^, ^80^, ^81^, ^82^, ^83^, ^84^, ^85^] Figure 10 shows the XRD structures of *trans*‐[PtCl_2_(NCNMe_2_)_2_]⋅2CHX_3_ (LIHMIB, X=Br and UKEKIG, X=I), which are almost equivalent to the palladium(II) assemblies shown in Figure 8. However, some subtle differences are worth commenting on. First, the Br⋅⋅⋅Pt and I⋅⋅⋅Pt distances are shorter than the Br⋅⋅⋅Pd and I⋅⋅⋅Pd contacts, suggesting that the Pt^II^ center is a better HaB *d_z_* ^2^ acceptor. Agreeably, the MEP surface plot represented in Figure 10(c) demonstrates that the MEP value above and below the platinum(II) center is larger in its absolute value than that at the palladium(II) atom (Figure 8c), and thus it is more suitable as an acceptor for the occurrence of σ‐hole interactions.

The QTAIM analysis of *trans*‐[PtCl_2_(NCNMe_2_)_2_] ⋅ 2CHI_3_ (Figure 10d) shows the corresponding bond CPs and bond paths connecting the I atoms to the Pt center, thus confirming the existence of this contact. The attractive nature of the interaction is evidenced by the NCIplot bluish isosurfaces located between the Pt^II^ and I‐centers. The NCI plot also demonstrates that the isosurface extends toward the NCNMe_2_ ligands, suggesting that the Pt‐bonded triple bond is also involved in the binding. The formation energy of the trimer (−15.1 kcal/mol) is larger (in absolute value) than that found for the LIHMAT structure (Figure 8), in good agreement with the MEP surface analysis (showing that Pt is more nucleophilic than Pd). Moreover, the I‐atom (UKEKIG) is better HaB donor than the Br‐atom (LIHMAT).

**Figure 11:**
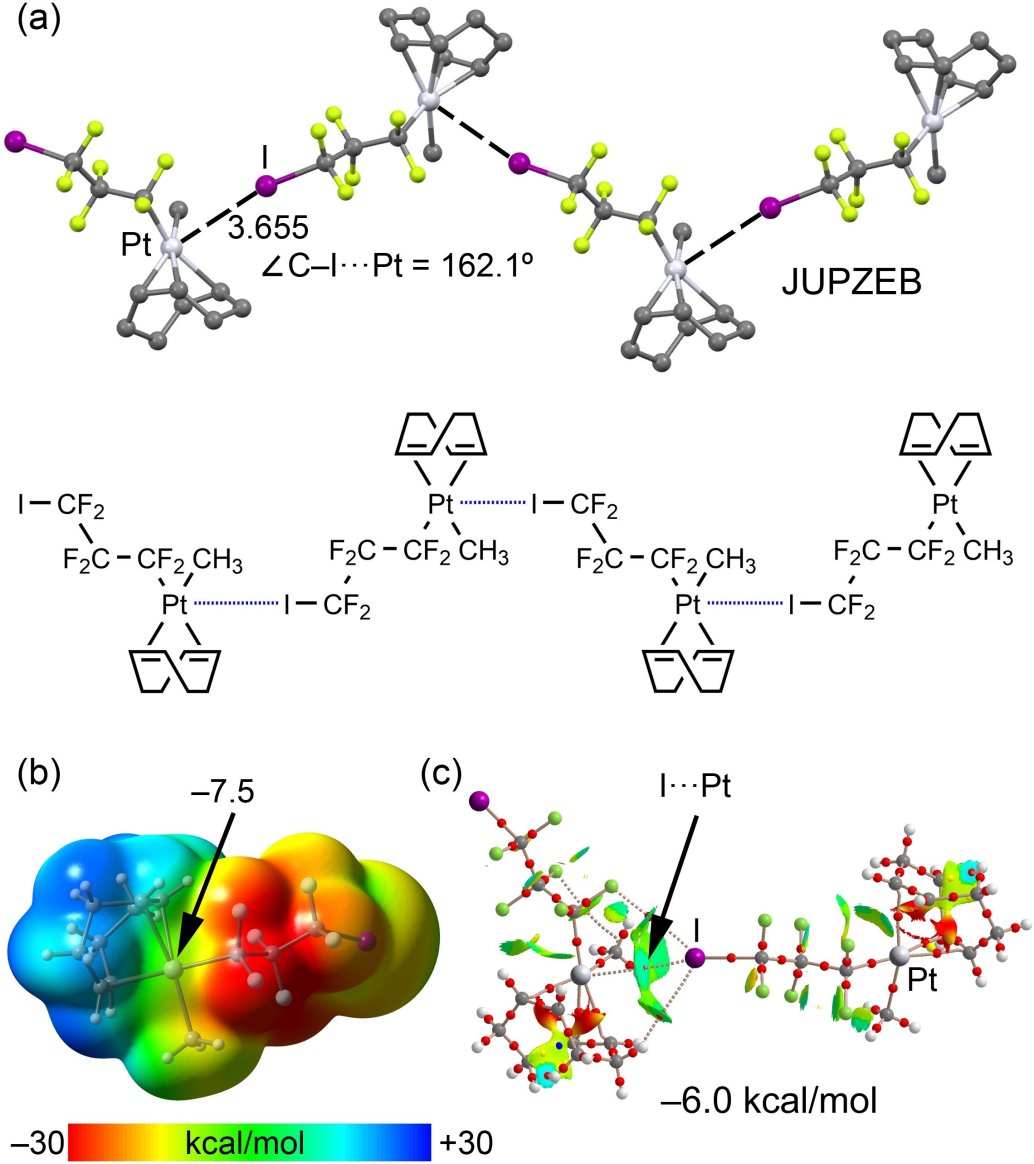
(a) Partial view of the XRD structure of CSD refcode JUPZEB.^[86]^ (b) MEP surface of the (COD)‐(perfluoro‐3‐iodopropyl)‐methyl‐platinum(II) complex using 0.001 a.u. The MEP values at selected points of the surface are indicated. (d) Combined QTAIM distribution of CPs (bond CPs in red and ring CPs in yellow) and bond paths with the NCIplot surfaces (isosurface = 0.5 a.u., gradient cutoff = 0.4 a.u., color scale −0.03 a.u. ≤ sign(*λ*_2_)*ρ* ≤ 0.03 a.u.) for a dimer of JUPZEB.^24^ The formation energy of the dimer is also indicated.

Another interesting structure^[86]^ is shown in Figure 11, which was obtained by the original authors to exclusively validate a mechanistic proposal; consequently, its solid‐state structure was not described. Remarkably, C−I⋅⋅⋅*d_z_* ^2^[Pt^II^] interactions propagate the structure into 1D‐supramolecular polymers (Figure 11a).

The I⋅⋅⋅Pt distance is longer than that observed in UKEKIG^[81]^ (Figure 10b), and the directionality is slightly worse, indirectly indicating a weaker interaction. The MEP surface of monomeric JUPZEB^[86]^ (Figure 11b) corroborates this fact, since the MEP value over the Pt atom is quite small, which is likely due to the effect of the H atoms of the cyclooctadiene ligand. The combined QTAIM/NCIPlot analysis verifies the existence of the C−I⋅⋅⋅*d_z_* ^2^[Pt^II^] short contact, which is characterized by a bond CP, a bond path and a green (attractive) NCIPlot isosurface. In addition to the C−I⋅⋅⋅*d_z_* ^2^[Pt^II^] contact, the QTAIM also reveals the existence of ancillary C−I⋅⋅⋅F and C−I⋅⋅⋅H interactions, which also contribute to the occurrence of the assembly. The dimerization energy is −6.0 kcal/mol, thus further supporting the higher ability of Pt compared to Ni or Pd to establish stronger σ‐hole⋅⋅⋅*d_z_* ^2^[M^II^] interactions.

**Figure 12:**
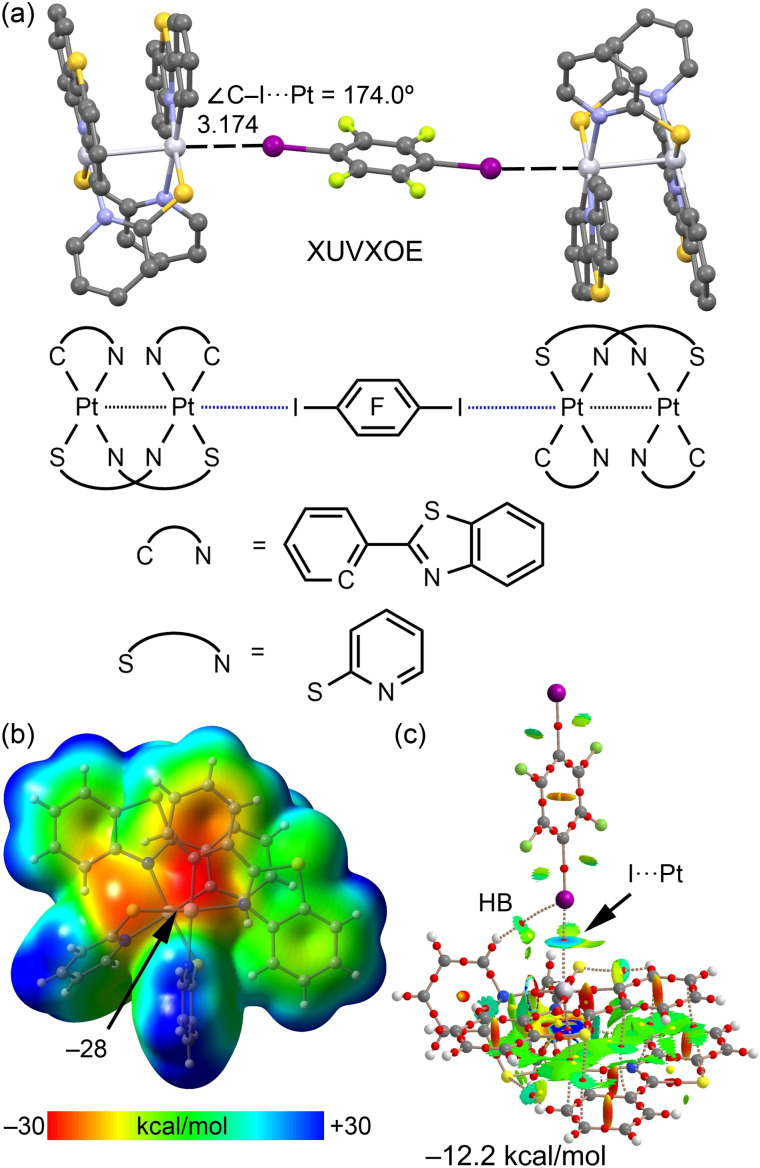
(a) Partial view of the XRD structure of CSD refcode XUVXOE.^[43]^ (b) MEP surface of the half‐lantern Pt^II^_2_ complex using the 0.001 a.u. The MEP values at selected points of the surface are indicated. (d) Combined QTAIM distribution of CPs (bond CPs in red and ring CPs in yellow) and bond paths with the NCIplot surfaces (isosurface = 0.5 a.u., gradient cutoff = 0.4 a.u., color scale −0.03 a.u. ≤ sign(*λ*_2_)*ρ* ≤ 0.03 a.u.). The formation energy of the dimer is also indicated.

Half‐lantern dinuclear Pt^II^_2_ complexes^[87]^ incorporating bridging (thio)azaheterocyclic ligands to provide rigidity to the system have recently been used to stimulate repulsive metal‐metal interactions between *d_z_* ^2^ orbitals of metal centers.^[43]^ This repulsion increases the nucleophilicity of the outer orbitals and consequently their ability to participate in HaB interactions. Figure 12(a) shows the XRD structure of one of the designed half‐lantern dinuclear platinum(II) complexes co‐crystallized with FIB (CSD refcode: XUVXOE),^[43]^ forming three component assemblies in the solid‐state, where the FIB molecule establishes symmetrically equivalent C−I⋅⋅⋅*d_z_* ^2^[Pt^II^] contacts, which are short and directional. A theoretical study has compared the MEP value over the Pt atom in the dinuclear complex (represented in Figure 12b) to that of an equivalent mononuclear complex, evidencing that in the dinuclear complex, the MEP value is 2.2 kcal/mol more negative, confirming that the nucleophilicity of the *d_z_* ^2^ orbital increases.^[43]^

This likely explains the short distance (3.174 versus 3.73 Å) and expressed directionality of the HaBs (174°) in the trimer. The QTAIM/NCI plot analysis shown in Figure 12(c) favors the enhanced ability of this type of system as an electron donor, revealing the blue color of the NCI plot isosurface in line with the strong interaction energy (−12.2 kcal/mol) that further supports the enhanced nucleophilicity of Pt^II^ with respect to other metal centers.

## 3 Conclusion

In this highlight, we critically analyzed our works focused on metal‐involving HaB. We also added several examples of HaB with metals that we revealed by searching and processing the Cambridge Structural Database; these examples were subject to theoretical calculations conducted in the framework of this minireview. Many examples of σ‐hole⋅⋅⋅*d_z_* ^2^[M] HaB interactions considered herein were unnoticed by the original authors, probably because this type of noncovalent bonding is counterintuitive and the nucleophilicity of positively charged metal centers was perceived as an oxymoron.

**Figure 13:**
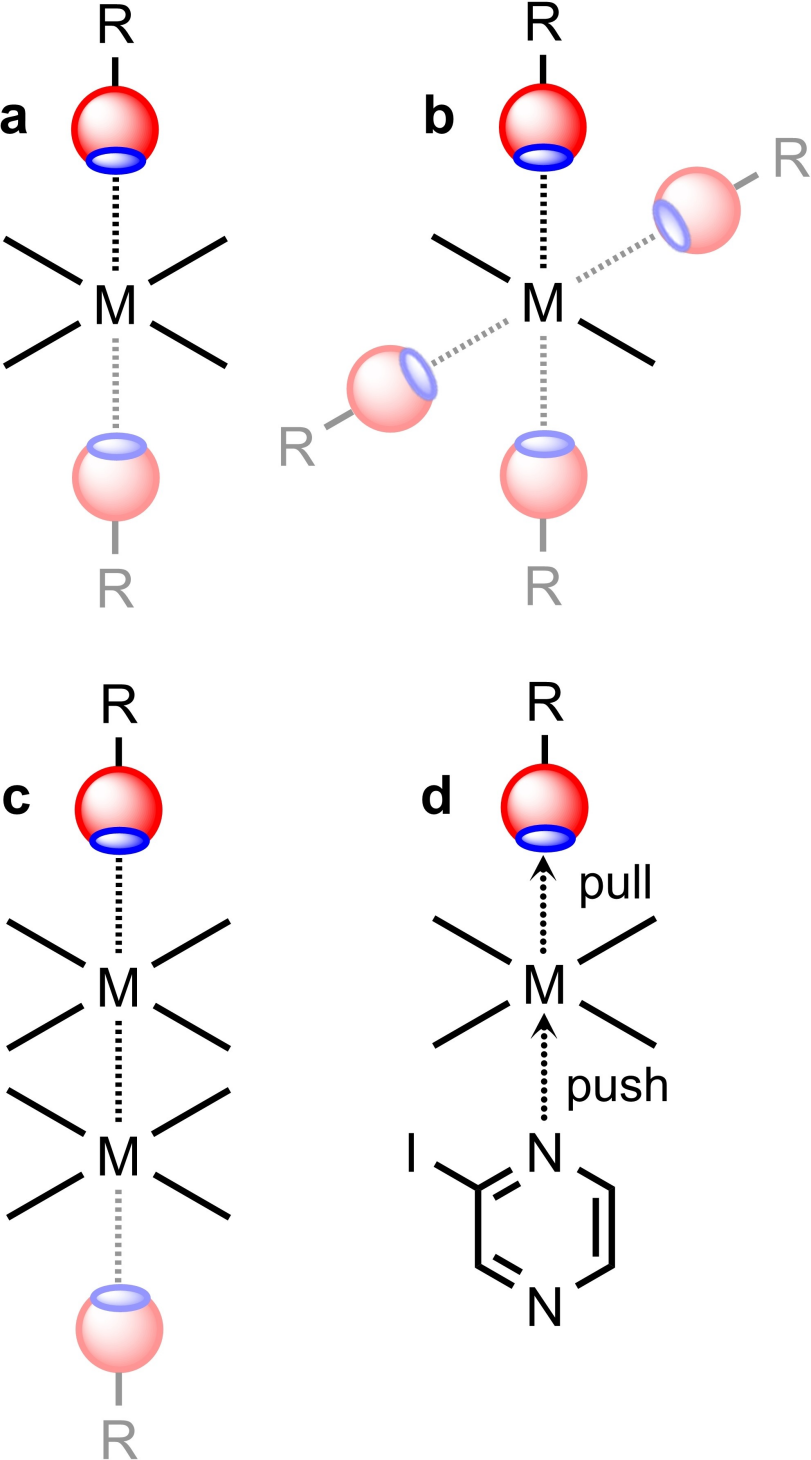
Types of interactions that lead to metal‐involving HaBs.

To date, few studies have purposefully utilized this interaction to design and construct supramolecular assemblies, where Rh^I^, Ni^II^, Pd^II^, and Pt^II^ complexes have been predominantly employed, and the maximum number of examples is listed for palladium‐ and platinum‐based systems. One can distinguish several types of interactions that lead to the metal‐involving HaBs: (i) binding of one (Figure 13a; full color HaB donor) or two (13a; full color plus semitone HaB donors) σ‐(Ha)‐hole donor(s) to metal orbital in the square‐planar surrounding; (ii) binding of one (Figure 13b; full color HaB donor) or four (13b; full color plus semitone HaB donors) σ‐(Ha)‐hole donor(s) to metal orbital of linear complexes; (iii) binding of one (Figure 13c; full color HaB donor) or two (13c; full color plus semitone HaB donors) σ‐(Ha)‐hole donor(s) to metal‐orbital of half‐lantern Pt_2_^II^ species; and (iv) σ‐hole⋅⋅⋅*d_z_* ^2^[M] interactions supported by the simultaneous push (iodine σ‐hole) and pull (electron donating center) nature of two noncovalent interacting partners (Figure 13d).

**Figure 14:**
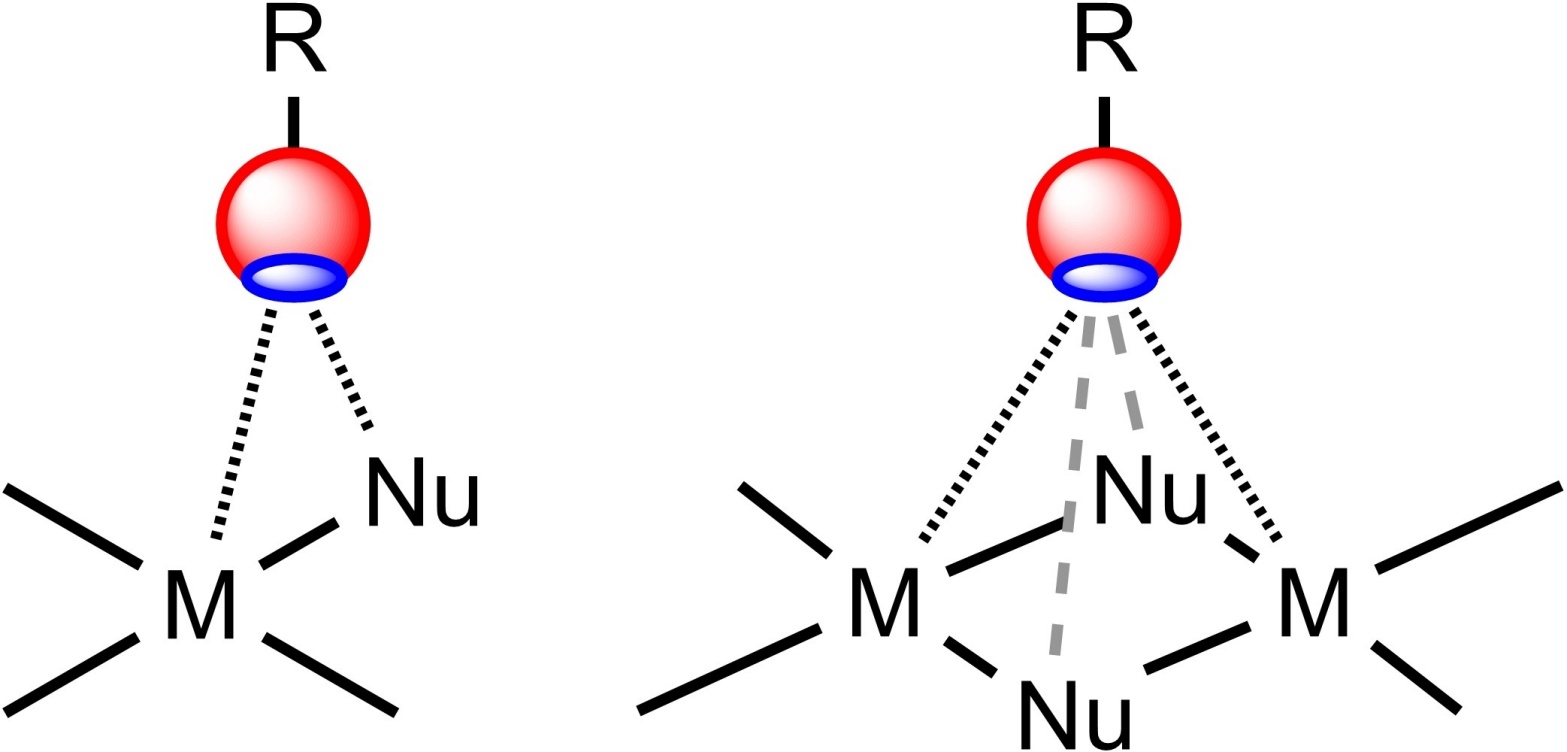
Integrated metal‐containing nucleophiles as components of multifurcated HaBs.

Notably, in the context of this consideration, the nucleophilic metal center could function alone or as an integrated two‐center Rh ^I^X_2_ Rh ^I^ (X=Cl, Br),^[46]^ Pt^II^−X (X=Cl,[^47^, ^81^] I[^85^, ^88^]), Pd^II^−C,^[43]^ (Figure 14; bold dotted lines) or even as a four‐center Rh^I^_2_Cl_2_ nucleophile^[46]^ (bold plus dashed lines). In these instances, HaB with a σ‐hole donor is simultaneously formed with those centers.

The perspectives of this minireview can be at least threefold. First, the occurrence of I−I⋅⋅⋅Pt^II^ HaB has been proposed as the initial stage of the oxidative addition of molecular iodine to a platinum(II) center.[^80^, ^82^, ^83^, ^89^, ^90^, ^91^] It is rather likely that the formation of similar C−X⋅⋅⋅Pd (X=Br, I) HaBs can be a key step in oxidative addition in C−C cross‐coupling reactions (Ref. [50,71] and the references therein). Thus, studies focused on metal‐involving HaBs can be significant for clarifying the fine mechanism of oxidative addition processes, including those of practical importance.

Second, apart from the metal‐involving HaB listed and verified in this highlight, positively charged metal centers can also function as nucleophiles toward other σ‐hole donating centers. In particular, we recently reported on chalcogen bond between Se or Te centers, functioning as σ‐hole donors, and Pt^II^‐based *d_z_* ^2^‐nucleophiles.^[92]^ The latter report is relevant to Lin and Gabbaï studies, in which the intramolecular interactions between Pd^II^ and telluronium centers^[93]^ and between Au^I^ and Te in telluroxide^[94]^ were identified. Another fraction of reports[^95^, ^96^, ^97^] focused on intramolecular pnictogen‐metal interactions. In modern terminology, all of these interactions could be attributed to metal‐involving chalcogen^[98]^ or pnictogen^[4]^ bonding. It is also noteworthy that metal‐involving HaB, uncovered in this minireview, is relevant to the well‐documented metal‐involving hydrogen bonding (for reviews see Ref. [99–102] and for recent studies see Ref. [103–107]).

Third, our recent studies[^95^, ^96^, ^97^] revealed *d* ^8^‐metal nucleophilicity toward π‐holes of electron‐deficient aromatic systems; for reviews comparing σ‐ and π‐donors of noncovalent interactions, see Ref. [34,108–110]

All facts listed in this section and their analysis indicate that the nucleophilic properties of *d* ^8^‐ and *d* ^10^‐centers as partners of noncovalent interactions may be common, regardless of the identity of electrophilic components. At this stage, metal‐involving HaB has been reliably established for 9–11 group metals, but we cannot not exclude that other electron reach metal centers (in particular, low‐oxidation state metal centers) of other periods can also function as nucleophilic components of HaB. We assume that these interactions will be increasingly used, and this minireview will stimulate additional interest in the diversity of chemistry fields that utilize metal‐involving noncovalent interactions.

## Conflict of interest

The authors declare no conflict of interest.

4

## Biographical Information

*Daniil M. Ivanov graduated (2014) and received his PhD degree in Chemistry (2017) at the Institute of Chemistry, Saint Petersburg State University, Russia. In 2021 he was appointed Associate Professor at the same Institute. His research interests include chemistry of late d‐metals, noncovalent interactions, and crystal engineering*.

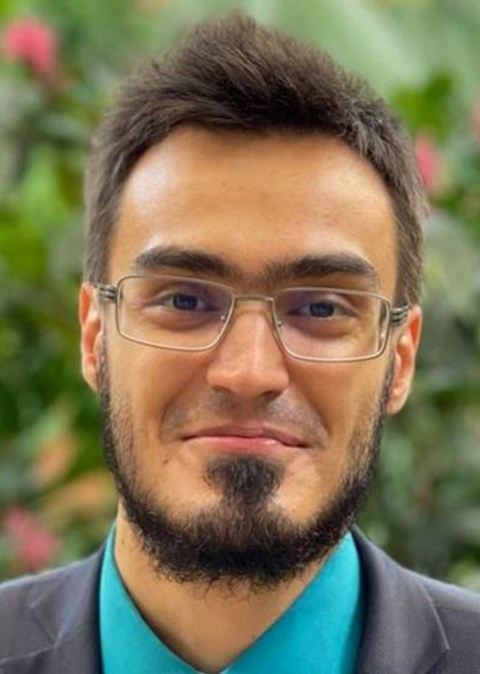

## Biographical Information

*Nadezhda A. Bokach graduated in Biology and Chemistry (1998) from Vologda State Pedagogical University, Russia. In 2002, she obtained her PhD degree in Chemistry from Saint Petersburg State Technological Institute (Technical University) and in 2012 she received her DSc degree (Organometallic Chemistry) from Saint Petersburg State University. Since 2015, she is Full Professor and RAS Professor at the Institute of Chemistry, Saint Petersburg State University. Her research interests include coordination chemistry, organometallic chemistry, ligand reactivity, metal‐involving organic synthesis, and noncovalent interactions*.

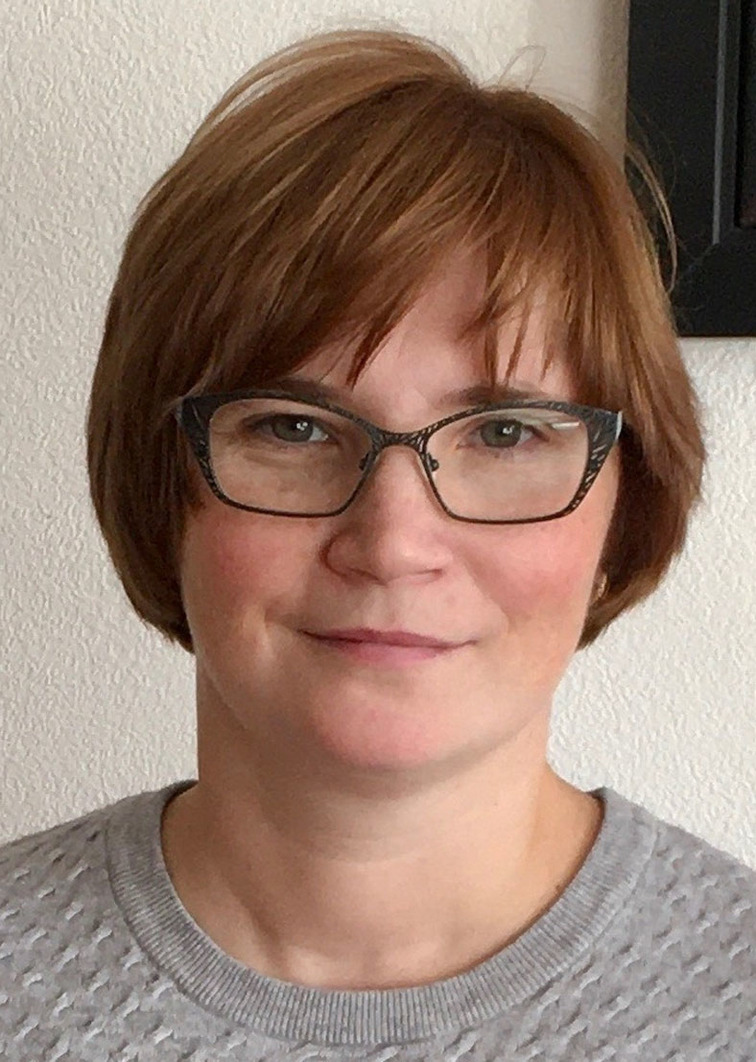

## Biographical Information

*Vadim Yurievich Kukushkin (born in 1956) studied chemistry at Lensovet Technological Institute (Technical University) and in 1984 he joined the faculty at Saint Petersburg State University. Currently he is a Full Member of the Russian Academy of Science and Full Professor of Saint Petersburg State University. His research interests include platinum group metal chemistry, ligand reactivity, noncovalent interactions, organic synthesis involving metal complexes, and catalysis. He is an author of ca. 400 original papers, patents, and reviews, as well as two books and a number of book chapters*.

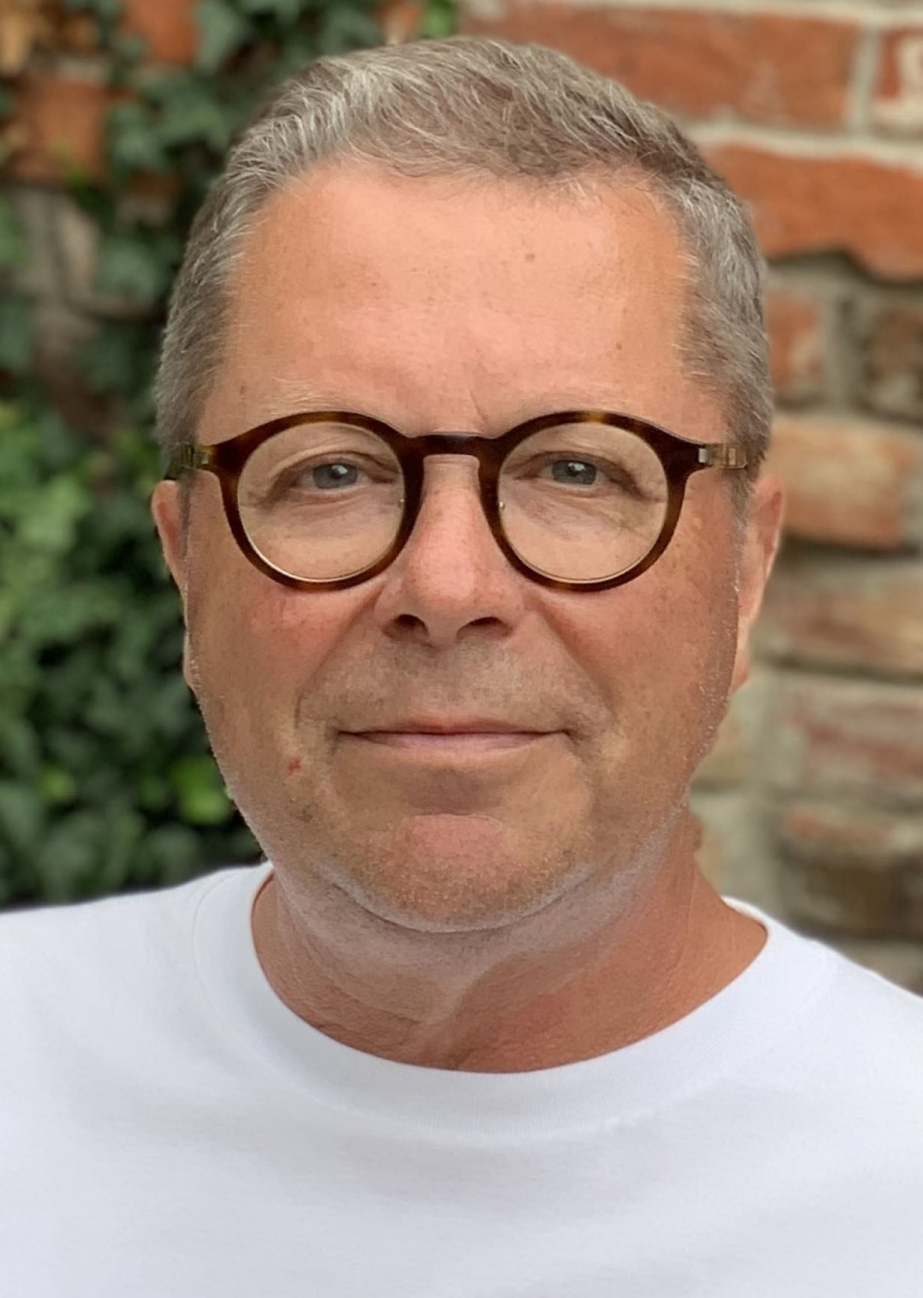

## Biographical Information

*Antonio Frontera (born in 1967) studied chemistry at the Department of Chemistry, Universitat de les Illes Balears, Spain. In 1995 and 1996 he had a postdoctoral stay at Yale University under the auspices of Prof. William L. Jorgensen. He is currently a Full Professor of Universitat de les Illes Balears. His research interests include supramolecular chemistry, noncovalent interactions, aromaticity and supramolecular catalysis. He is an author of ca. 700 original papers, and reviews, as well as a number of book chapters*.

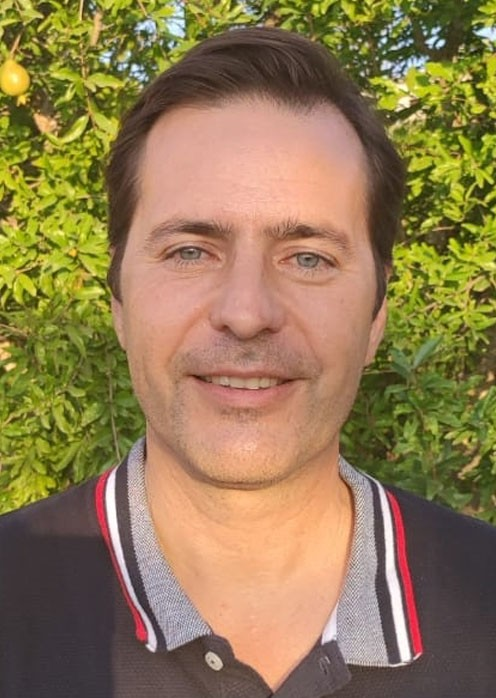

## Data Availability

The data that support the findings of this study are available from the corresponding author upon reasonable request.

## References

[1] G. Cavallo , P. Metrangolo , R. Milani , T. Pilati , A. Priimagi , G. Resnati , G. Terraneo , Chem. Rev. 2016, 116, 2478–2601. 26812185 10.1021/acs.chemrev.5b00484 PMC4768247

[2] P. Politzer , J. S. Murray , Crystals 2019, 9.

[3] G. Berger , P. Frangville , F. Meyer , Chem. Commun. 2020, 56, 4970–4981. 10.1039/d0cc00841a 32297598

[4] L. Brammer , Faraday Discuss. 2017, 203, 485–507. 28980683 10.1039/c7fd00199a

[5] B. Li , S. Q. Zang , L. Y. Wang , T. C. W. Mak , Coord. Chem. Rev. 2016, 308, 1–21.

[6] A. M. Maharramov , K. T. Mahmudov , M. N. Kopylovich , A. J. Pombeiro , in Non-covalent Interactions in the Synthesis and Design of New Compounds, Wiley Online Library, 2016.

[7] J. Pancholi , P. D. Beer , Coord. Chem. Rev. 2020, 416.

[8] A. Mukherjee , S. Tothadi , G. R. Desiraju , Acc. Chem. Res. 2014, 47, 2514–2524. 25134974 10.1021/ar5001555

[9] R. Tepper , U. S. Schubert , Angew. Chem. Int. Ed. 2018, 57, 6004–6016. 10.1002/anie.201707986 29341377

[10] J. Y. C. Lim , P. D. Beer , Chem 2018, 4, 731–783.

[11] S. Scheiner , M. Michalczyk , W. Zierkiewicz , Coord. Chem. Rev. 2020, 405.

[12] F. Heinen , D. L. Reinhard , E. Engelage , S. M. Huber , Angew. Chem. Int. Ed. 2021, 60, 5069–5073. 10.1002/anie.202013172 PMC7986438 33215804

[13] R. L. Sutar , S. M. Huber , ACS Catal. 2019, 9, 9622–9639.

[14] S. H. Jungbauer , S. M. Huber , J. Am. Chem. Soc. 2015, 137, 12110–12120. 26329271 10.1021/jacs.5b07863

[15] A. Dreger , P. Wonner , E. Engelage , S. M. Walter , R. Stoll , S. M. Huber , Chem. Commun. 2019, 55, 8262–8265. 10.1039/c9cc02816a 31243400

[16] K. T. Mahmudov , A. V. Gurbanov , F. I. Guseinov , M. F. C. Guedas da Silva , Coord. Chem. Rev. 2019, 387, 32–46.

[17] S. E. Shetgaonkar , F. V. Singh , Front. Chem. 2020, 8, 705. 33134246 10.3389/fchem.2020.00705 PMC7553084

[18] Y. Shigeyuki , K. Tsutomu , Curr. Org. Chem. 2020, 24, 2118–2152.

[19] K. T. Mahmudov, M. N. Kopylovich, M. F. C. Guedes da Silva, A. J. L. Pombeiro, *Noncovalent Interactions in Catalysis*, The Royal Society of Chemistry, **2019**.

[20] P. Nagorny , Z. K. Sun , Beilstein J. Org. Chem. 2016, 12, 2834–2848. 28144357 10.3762/bjoc.12.283 PMC5238598

[21] D. Bulfield , S. M. Huber , Chem. Eur. J. 2016, 22, 14434–14450. 27465662 10.1002/chem.201601844

[22] S. Benz , A. I. Poblador-Bahamonde , N. Low-Ders , S. Matile , Angew. Chem. Int. Ed. 2018, 57, 5408–5412. 10.1002/anie.201801452 PMC5947745 29558562

[23] M. Breugst , D. von der Heiden , J. Schmauck , Synthesis 2017, 49, 3224–3236.

[24] K. T. Mahmudov , M. N. Kopylovich , M. F. C. Guedas da Silva , A. J. L. Pombeiro , Coord. Chem. Rev. 2017, 345, 54–72.

[25] G. Berger , J. Soubhye , F. Meyer , Polym. Chem. 2015, 6, 3559–3580.

[26] L. Mendez , G. Henriquez , S. Sirimulla , M. Narayan , Molecules 2017, 22, 1397. 10.3390/molecules22091397 PMC6151711 28837116

[27] P. S. Ho , Future Med. Chem. 2017, 9, 637–640. 28485645 10.4155/fmc-2017-0052

[28] A. Dalpiaz , B. Pavan , V. Ferretti , Drug Discovery Today 2017, 22, 1134–1138. 28130117 10.1016/j.drudis.2017.01.010

[29] Y. X. Lu , Y. T. Liu , Z. J. Xu , H. Y. Li , H. L. Liu , W. L. Zhu , EODDBX 2012, 7, 375–383.

[30] C. A. Bayse , New J. Chem. 2018, 42, 10623–10632. 10.1039/C8NJ00557E PMC6376990 30778278

[31] A. M. S. Riel , R. K. Rowe , E. N. Ho , A. C. C. Carlsson , A. K. Rappe , O. B. Berryman , P. S. Ho , Acc. Chem. Res. 2019, 52, 2870–2880. 31318520 10.1021/acs.accounts.9b00189 PMC7328900

[32] J. Teyssandier , K. S. Mali , S. De Feyter , ChemistryOpen 2020, 9, 225–241. 32071832 10.1002/open.201900337 PMC7011184

[33] D. Bulfield , E. Engelage , L. Mancheski , J. Stoesser , S. M. Huber , Chem. Eur. J. 2020, 26, 1567–1575. 31638284 10.1002/chem.201904322 PMC7028063

[34] A. Bauza , T. J. Mooibroek , A. Frontera , ChemPhysChem 2015, 16, 2496–2517. 26118675 10.1002/cphc.201500314

[35] S. K. Seth , A. Bauzá , A. Frontera , in Understanding Intermolecular Interactions in the Solid State: Approaches and Techniques, Royal Soc. Chem., 2019, pp. 285–333.

[36] J. Frosch , M. Koneczny , T. Bannenberg , M. Tamm , Chem. Eur. J. 2021, 27, 4349–4363. 33094865 10.1002/chem.202004418 PMC7986712

[37] A. S. Mikherdov , A. S. Novikov , V. P. Boyarskiy , V. Y. Kukushkin , Nat. Commun. 2020, 11, 2921. 32523100 10.1038/s41467-020-16748-x PMC7286913

[38] J. E. Del Bene , I. Alkorta , J. Elguero , Molecules 2017, 22, 1955. 10.3390/molecules22111955 PMC6150174 29137153

[39] A. N. Isaev , Chem. Phys. Lett. 2021, 763, 138195. 10.1016/j.cplett.2020.138193 PMC7666712 33223560

[40] Y. V. Torubaev , K. A. Lyssenko , P. Y. Barzilovich , G. A. Saratov , M. M. Shaikh , A. Singh , P. Mathur , CrystEngComm 2017, 19, 5114–5121.

[41] Y. V. Torubaev , I. V. Skabitsky , G. A. Saratov , P. Y. Barzilovich , Mendeleev Commun. 2021, 31, 58–61.

[42] N. Ramanathan , K. Sundararajan , K. Vidya , E. D. Jemmis , Spectrochim. Acta A Mol. Biomol. Spectrosc. 2016, 157, 69–78. 26722673 10.1016/j.saa.2015.12.016

[43] E. A. Katlenok , M. Haukka , O. V. Levin , A. Frontera , V. Y. Kukushkin , Chem. Eur. J. 2020, 26, 7692–7701. 32240560 10.1002/chem.202001196

[44] Z. M. Bikbaeva , D. M. Ivanov , A. S. Novikov , I. V. Ananyev , N. A. Bokach , V. Y. Kukushkin , Inorg. Chem. 2017, 56, 13562–13578. 29068669 10.1021/acs.inorgchem.7b02224

[45] G. R. Desiraju , P. S. Ho , L. Kloo , A. C. Legon , R. Marquardt , P. Metrangolo , P. Politzer , G. Resnati , K. Rissanen , Pure Appl. Chem. 2013, 85, 1711.

[46] A. A. Eliseeva , D. M. Ivanov , A. V. Rozhkov , I. V. Ananyev , A. Frontera , V. Y. Kukushkin , JACS Au 2021, 1, 354–361. 34467299 10.1021/jacsau.1c00012 PMC8395620

[47] U. Dabranskaya , D. M. Ivanov , A. S. Novikov , Y. V. Matveychuk , N. A. Bokach , V. Y. Kukushkin , Cryst. Growth Des. 2019, 19, 1364–1376.

[48] A. S. Novikov , Inorg. Chim. Acta 2018, 471, 126–129.

[49] A. V. Rozhkov , D. M. Ivanov , A. S. Novikov , I. V. Ananyev , N. A. Bokach , V. Y. Kukushkin , CrystEngComm 2020, 22, 554–563.

[50] L. E. Zelenkov , A. A. Eliseeva , S. V. Baykov , V. V. Suslonov , B. Galmés , A. Frontera , V. Y. Kukushkin , D. M. Ivanov , N. A. Bokach , Inorg. Chem. Front. 2021.

[51] M. Freindorf , S. Yannacone , V. Oliveira , N. Verma , E. Kraka , Crystals 2021, 11, 373.

[52] A. Y. Rogachev , R. Hoffmann , J. Am. Chem. Soc. 2013, 135, 3262–3275. 23384185 10.1021/ja312584u

[53] F. Groenewald , J. Dillen , C. Esterhuysen , New J. Chem. 2018, 42, 10529–10538.

[54] R. Y. Liau , H. Ehlich , A. Schier , H. Schmidbaur , Z. Naturforsch. Sect. B - J. Chem. Sci. 2002, 57, 1085–1089.

[55] H. M. Yamamoto , J. I. Yamaura , R. Kato , J. Am. Chem. Soc. 1998, 120, 5905–5913.

[56] D. Schneider , A. Schier , H. Schmidbaur , Dalton Trans. 2004, 1995–2005. 15252587 10.1039/b403005b

[57] I. Blakey , Z. Merican , L. Rintoul , Y. M. Chuang , K. S. Jack , A. S. Micallef , Phys. Chem. Chem. Phys. 2012, 14, 3604–3611. 22314792 10.1039/c2cp23809h

[58] R. N. Dhital , C. Kamonsatikul , E. Somsook , Y. Sato , H. Sakurai , Chem. Commun. 2013, 49, 2542–2544. 10.1039/c3cc39019e 23423533

[59] P. Maity , K. Sasai , R. N. Dhital , H. Sakai , T. Hasobe , H. Sakurai , J. Phys. Chem. Lett. 2020, 11, 1199–1203. 31967476 10.1021/acs.jpclett.9b03557

[60] Y. Komoto , S. Fujii , K. Hara , M. Kiguchi , J. Phys. Chem. C 2013, 117, 24277–24282.

[61] L. L. Peng , B. Huang , Q. Zou , Z. W. Hong , J. F. Zheng , Y. Shao , Z. J. Niu , X. S. Zhou , H. J. Xie , W. B. Chen , Nanoscale Res. Lett. 2018, 13, 121. 29808266 10.1186/s11671-018-2528-z PMC5972139

[62] P. S. Cheng , S. C. K. Hau , T. C. W. Mak , Austr. J. Chem. 2014, 67, 1849–1859.

[63] Y. Morishima , D. J. Young , K. Fujisawa , Dalton Trans. 2014, 43, 15915–15928. 25230795 10.1039/c4dt01978d

[64] G. Yang , P. Baran , A. R. Martinez , R. G. Raptis , Cryst. Growth Des. 2013, 13, 264–269.

[65] W. M. Bloch , C. J. Sumby , Supramol. Chem. 2015, 27, 807–819.

[66] O. I. Kucheriv , S. I. Shylin , V. Ksenofontov , S. Dechert , M. Haukka , I. O. Fritsky , I. A. Gural′skiy , Inorg. Chem. 2016, 55, 4906–4914. 27120049 10.1021/acs.inorgchem.6b00446

[67] A. V. Rozhkov , A. A. Eliseeva , S. V. Baykov , B. Galmes , A. Frontera , V. Y. Kukushkin , Cryst. Growth Des. 2020, 20, 5908–5921.

[68] D. W. Shaffer , S. A. Ryken , R. A. Zarkesh , A. F. Heyduk , Inorg. Chem. 2012, 51, 12122–12131. 24490662 10.1021/ic300733j

[69] L. Carreras , J. Benet-Buchholz , A. Franconetti , A. Frontera , P. van Leeuwen , A. Vidal-Ferran , Chem. Commun. 2019, 55, 2380–2383. 10.1039/c8cc08884e 30729960

[70] S. S. Batsanov , Inorg. Mater. 2001, 37, 871–885.

[71] S. V. Baykov , U. Dabranskaya , D. M. Ivanov , A. S. Novikov , V. P. Boyarskiy , Cryst. Growth Des. 2018, 18, 5973–5980.

[72] P. Heines , H. L. Keller , M. Armbruster , U. Schwarz , J. Tse , Inorg. Chem. 2006, 45, 9818–9825. 17112279 10.1021/ic0601945

[73] P. Heines , H. L. Keller , P. Bouvier , M. Hanfland , Z. Anorg. Allg. Chem. 2003, 629, 545–550.

[74] B. Schüpp , P. Heines , H.-L. Keller , Z. Anorg. Allg. Chem. 2000, 626, 202–207.

[75] B. Schupp , P. Heines , A. Savin , H. L. Keller , Inorg. Chem. 2000, 39, 732–735. 11272568 10.1021/ic990670+

[76] Y. Yamashina , Y. Kataoka , Y. Ura , Eur. J. Inorg. Chem. 2014, 4073–4078. 10.1021/ic403050c 24661117

[77] Y. Yamashina , Y. Kataoka , Y. Ura , Inorg. Chem. 2014, 53, 3558–3567. 24661117 10.1021/ic403050c

[78] E. A. Katlenok , A. V. Rozhkov , O. V. Levin , M. Haukka , M. L. Kuznetsov , V. Y. Kukushkin , Cryst. Growth Des. 2021, 21, 1159–1177.

[79] J. L. Serrano , L. Garcia , J. Perez , E. Perez , J. Garcia , G. Sanchez , P. Sehnal , S. De Ornellas , T. J. Williams , I. J. S. Fairlamb , Organometallics 2011, 30, 5095–5109.

[80] R. A. Gossage , A. D. Ryabov , A. L. Spek , D. J. Stufkens , J. A. M. van Beek , R. van Eldik , G. van Koten , J. Am. Chem. Soc. 1999, 121, 2488–2497.

[81] D. M. Ivanov , A. S. Novikov , I. V. Ananyev , Y. V. Kirina , V. Y. Kukushkin , Chem. Commun. 2016, 52, 5565–5568. 10.1039/c6cc01107a 27020251

[82] J. A. M. van Beek , G. van Koten , G. P. C. M. Dekker , E. Wissing , M. C. Zoutberg , C. H. Stam , J. Organomet. Chem. 1990, 394, 659–678.

[83] J. A. M. van Beek , G. van Koten , W. J. J. Smeets , A. L. Spek , J. Am. Chem. Soc. 1986, 108, 5010–5011.

[84] L. S. von Chrzanowski , M. Lutz , A. L. Spek , B. Suijkerbuijk , R. Gebbink , Acta Crystallogr. Sect. E 2007, 63, M1223-M1225.

[85] M. Bulatova , D. M. Ivanov , M. Haukka , Cryst. Growth Des. 2021, 21, 974–987.

[86] L. Xu , D. P. Solowey , D. A. Vicic , Organometallics 2015, 34, 3474–3479.

[87] M. Chaaban , C. K. Zhou , H. R. Lin , B. Chyi , B. W. Ma , J. Mater. Chem. C 2019, 7, 5910–5924.

[88] M. Bulatova , D. M. Ivanov , J. M. Rautiainen , M. A. Kinzhalov , K.-N. Truong , M. Lahtinen , M. Haukka , Inorg. Chem. 2021, 60, 13200–13211. 10.1021/acs.inorgchem.1c01591 PMC8424624 34357775

[89] D. Hopgood , R. A. Jenkins , J. Am. Chem. Soc. 1973, 95, 4461–4463.

[90] P. M. Cook , L. F. Dahl , D. Hopgood , R. A. Jenkins , J. Chem. Soc. Dalton Trans. 1973, 294–301.

[91] L. S. von Chrzanowski , M. Lutz , A. L. Spek , B. M. J. M. Suijkerbuijk , R. J. M. Klein Gebbink , Acta Crystallogr. Sect. E 2007, 63, m1223-m1225.

[92] A. V. Rozhkov , E. A. Katlenok , M. V. Zhmykhova , A. Y. Ivanov , M. L. Kuznetsov , N. A. Bokach , V. Y. Kukushkin , J. Am. Chem. Soc. 2021, 143, 15701–15710. 34529411 10.1021/jacs.1c06498

[93] T.-P. Lin , F. P. Gabbaï , Angew. Chem. Int. Ed. 2013, 52, 3864–3868. 10.1002/anie.201300337 23417975

[94] H. Yang , T.-P. Lin , F. P. Gabbaï , Organometallics 2014, 33, 4368–4373.

[95] A. V. Rozhkov , M. A. Krykova , D. M. Ivanov , A. S. Novikov , A. A. Sinelshchikova , M. V. Volostnykh , M. A. Konovalov , M. S. Grigoriev , Y. G. Gorbunova , V. Y. Kukushkin , Angew. Chem. Int. Ed. 2019, 58, 4164–4168. 10.1002/anie.201814062 30667579

[96] S. V. Baykov , S. I. Filimonov , A. V. Rozhkov , A. S. Novikov , I. V. Ananyev , D. M. Ivanov , V. Y. Kukushkin , Cryst. Growth Des. 2020, 20, 995–1008.

[97] A. V. Rozhkov , I. V. Ananyev , R. M. Gomila , A. Frontera , V. Y. Kukushkin , Inorg. Chem. 2020, 59, 9308–9314. 32516531 10.1021/acs.inorgchem.0c01170

[98] C. B. Aakeroy , D. L. Bryce , G. R. Desiraju , A. Frontera , A. C. Legon , F. Nicotra , K. Rissanen , S. Scheiner , G. Terraneo , P. Metrangolo , G. Resnati , Pure Appl. Chem. 2019, 91, 1889–1892.

[99] H. G. Raubenheimer , L. Dobrzańska , Coord. Chem. Rev. 2020, 402, 213052.

[100] L. Brammer , Dalton Trans. 2003, 3145–3157.

[101] T. Steiner , Angew. Chem. Int. Ed. 2002, 41, 48–76.

[102] E. R. T. Tiekink , Coord. Chem. Rev. 2017, 345, 209–228.

[103] H. Darmandeh , J. Löffler , N. V. Tzouras , B. Dereli , T. Scherpf , K.-S. Feichtner , S. Vanden Broeck , K. Van Hecke , M. Saab , C. S. J. Cazin , L. Cavallo , S. P. Nolan , V. H. Gessner , Angew. Chem. Int. Ed. 2021, 60, 21014–21024. 10.1002/anie.202108581 PMC8518757 34313367

[104] G. Park , F. P. Gabbaï , J. Am. Chem. Soc. 2021, 143, 12494–12498. 34369751 10.1021/jacs.1c07035

[105] M. Kumar , J. S. Francisco , J. Am. Chem. Soc. 2020, 142, 6001–6006. 32126169 10.1021/jacs.9b05493

[106] H. Schmidbaur , Angew. Chem. Int. Ed. 2019, 58, 5806–5809. 10.1002/anie.201902526 30941857

[107] P. Wang , H.-G. Xu , G.-J. Cao , W.-J. Zhang , X.-L. Xu , W.-J. Zheng , J. Phys. Chem. A 2017, 121, 8973–8981. 29088541 10.1021/acs.jpca.7b09428

[108] H. Wang , W. Wang , W. J. Jin , Chem. Rev. 2016, 116, 5072–5104. 26886515 10.1021/acs.chemrev.5b00527

[109] A. S. Mahadevi , G. N. Sastry , Chem. Rev. 2016, 116, 2775–2825. 26840650 10.1021/cr500344e

[110] J. S. Murray , P. Lane , T. Clark , K. E. Riley , P. Politzer , J. Mol. Model. 2012, 18, 541–548. 21541742 10.1007/s00894-011-1089-1

